# Dietary carbohydrate intake is associated with the subgingival plaque oral microbiome abundance and diversity in a cohort of postmenopausal women

**DOI:** 10.1038/s41598-022-06421-2

**Published:** 2022-02-16

**Authors:** Amy E. Millen, Runda Dahhan, Jo L. Freudenheim, Kathleen M. Hovey, Lu Li, Daniel I. McSkimming, Chris A. Andrews, Michael J. Buck, Michael J. LaMonte, Keith L. Kirkwood, Yijun Sun, Vijaya Murugaiyan, Maria Tsompana, Jean Wactawski-Wende

**Affiliations:** 1 Department of Epidemiology and Environmental Health, School of Public Health and Health Professions, University at Buffalo, 270 Farber Hall, Buffalo, NY 14214-8001 USA; 2 Department of Oral Biology, School of Dental Medicine, University at Buffalo, Buffalo, NY USA; 3 Division of Infectious Disease & International Medicine, Morsani College of Medicine, University of South Florida, Tampa, FL USA; 4 Department of Ophthalmology & Visual Sciences, University of Michigan, Ann Arbor, MI USA; 5 Department of Biochemistry, Jacobs School of Medicine and Biomedical Sciences, University at Buffalo, Buffalo, NY USA; 6 Department of Microbiology and Immunology, Jacobs School of Medicine and Biomedical Sciences, University at Buffalo, Buffalo, NY USA

**Keywords:** Carbohydrates, Bacteria, Microbial communities, Oral diseases

## Abstract

Limited research exists on carbohydrate intake and oral microbiome diversity and composition assessed with next-generation sequencing. We aimed to better understand the association between habitual carbohydrate intake and the oral microbiome, as the oral microbiome has been associated with caries, periodontal disease, and systemic diseases. We investigated if total carbohydrates, starch, monosaccharides, disaccharides, fiber, or glycemic load (GL) were associated with the diversity and composition of oral bacteria in subgingival plaque samples of 1204 post-menopausal women. Carbohydrate intake and GL were assessed from a food frequency questionnaire, and adjusted for energy intake. The V3–V4 region of the 16S rRNA gene from subgingival plaque samples were sequenced to identify the relative abundance of microbiome compositional data expressed as operational taxonomic units (OTUs). The abundance of OTUs were centered log(2)-ratio transformed to account for the compositional data structure. Associations between carbohydrate/GL intake and microbiome alpha-diversity measures were examined using linear regression. PERMANOVA analyses were conducted to examine microbiome beta-diversity measures across quartiles of carbohydrate/GL intake. Associations between intake of carbohydrates and GL and the abundance of the 245 identified OTUs were examined by using linear regression. Total carbohydrates, GL, starch, lactose, and sucrose intake were inversely associated with alpha-diversity measures. Beta-diversity across quartiles of total carbohydrates, fiber, GL, sucrose, and galactose, were all statistically significant (p for PERMANOVA p < 0.05). Positive associations were observed between total carbohydrates, GL, sucrose and *Streptococcus mutans;* GL and both *Sphingomonas HOT 006* and *Scardovia wiggsiae;* and sucrose and *Streptococcus lactarius.* A negative association was observed between lactose and *Aggregatibacter segnis,* and between sucrose and both *TM7_[G-1] HOT 346* and *Leptotrichia HOT 223*. Intake of total carbohydrate, GL, and sucrose were inversely associated with subgingival bacteria alpha-diversity, the microbial beta-diversity varied by their intake, and they were associated with the relative abundance of specific OTUs. Higher intake of sucrose, or high GL foods, may influence poor oral health outcomes (and perhaps systemic health outcomes) in older women via their influence on the oral microbiome.

## Introduction

The human microbiome plays a critical role in human health and disease^1^. In particular, the oral microbiome is associated with not only the health of the mouth, but also risk of other chronic diseases (e.g., cardiovascular disease^2,3^ hypertension^4^, type 2 diabetes^5^, and cancer^6,7^). Understanding of the factors (e.g., dietary intake, smoking behavior, medication use, etc.) affecting the composition of the oral microbiome is critical to understanding these observed associations with disease outcomes.

Over 700 different species of bacteria have been identified in the oral cavity^8^ with, on average, more than 250 different species in any one individual mouth^9^. The diversity of the oral microbiome in relation to oral health is complex. For example, previous data shows that the alpha-diversity of the microbiome in supragingival plaque samples (where cariogenic pathogens reside), decreases with the severity of caries^10^. Differently, the alpha-diversity in subgingival plaque samples, (where periodontal pathogens reside), increases with increasing severity of periodontal disease^11,12^, and such a relationship was observed in this cohort with the microbiome of our subgingival plaque samples^13^.

Diet has been shown to be associated with both caries and periodontal disease^14^ and hypothesized to influence the microbial composition and diversity of the saliva and gingival crevicular fluid^15^. Fermentable carbohydrates (simple sugars and starch) are significant sources of bacterial energy metabolism and are broken down by both bacterial enzymes and by endogenous processes in the oral cavity^15^. There is evidence that fermentable carbohydrates are essential to development of dental caries^16^. However, the association of carbohydrate intake with periodontal disease is less well studied^17–21^. Few studies have examining habitual intake of dietary carbohydrates in relation to the diversity and composition of the oral microbiome^22–24^.

We studied the association between habitual dietary carbohydrate intake and the subgingival plaque oral microbiome in a cohort of 1204 postmenopausal women, using data from the Buffalo Osteoporosis and Periodontal Disease (OsteoPerio) Study, a cohort study ancillary to the Women’s Health Initiative (WHI) Observational Study (OS)^25^. The OsteoPerio Study used 16S rRNA gene sequencing of oral plaque samples to identify and measure the relative abundance of the oral bacteria found^26^. We hypothesized that the alpha-diversity (within-subject diversity [number of species]) of the oral microbiome would be associated with intake of total carbohydrates, GL, starch, disaccharides (lactose, maltose, sucrose) and monosaccharides (fructose, galactose, and glucose) and that the beta-diversity (between group diversity) of the oral microbiome would differ across quartiles of intake in all carbohydrates and glycemic load (GL).

## Methods

### Study design

The OsteoPerio Study is an ongoing prospective cohort^26^, and ancillary to the WHI, a national study focused on health outcomes of postmenopausal women^25^. The OsteoPerio study was originated to examine the association between osteoporosis and loss of bone in the oral cavity^27^. Study participants were recruited from the WHI clinical center in Buffalo, NY between 1997 and 2001; 1,342 women participated in the baseline exam (Supplemental Fig. 1)^26^. Women were excluded if they had fewer than 6 teeth, bilateral hip replacement, a history of non-osteoporotic bone disease, a recent 10 years history of cancer, or if they were treated for serious diseases^26^. There were 1222 women with sequenced subgingival microbiome and dietary data at baseline, and of these, 18 women were excluded because their self-reported energy intakes were > 5000 or < 600 kcals, leaving a sample of 1204 women. All participants provided informed consent, and the study protocol was approved by the University at Buffalo’s Health Sciences Institutional Review Board. All experiments were in agreement with relevant guidelines regarding Human Subjects Research.

### Assessment of dietary carbohydrate intake

Dietary intake was assessed as part of the WHI OS participant’s year 3 visit, which coincided with the OsteoPerio baseline exam^26^. A modified Block food frequency questionnaire (FFQ), with 122 main questions and 4 summary questions, was administered to participants asking them to recall usual consumption during the last 3 months^28^. The WHI FFQ has been validated in a study conducted among 113 women in the WHI comparing the FFQ to mean intake from four, 24-h recalls and one 4-day food record^28^. The energy-adjusted Pearson correlation coefficients for total carbohydrates and total fiber were 0.63 and 0.65, respectively^28^. Our main exposures include intake of total carbohydrates, GL, total fiber, soluble fiber, insoluble fiber, starch, disaccharide intake (lactose, maltose, sucrose) and monosaccharide intake (fructose, galactose, and glucose). GL reflects both the amount of carbohydrate in a food in addition to its influence on blood sugar. In this study, total carbohydrate including fiber intake, rather than available carbohydrate intake, was used to estimate the GL^29^. Carbohydrate intake is presented as the percent of calories from carbohydrate consumed, or in the case of fiber intake and GL, as grams per 1,000 kcals consumed. All analyses used these energy-adjusted variables.

### Subgingival plaque samples and sequencing

A dental examiner performed an oral examination wherein subgingival plaque samples were taken with paper points from 12 index teeth (or substitutes), as described previously^30^. Paper points were inserted into subgingival pockets of a tooth’s mesiobuccal surface, with samples taken from maxillary and mandibular teeth and stored in freezers at − 80 °C.

The composition and diversity of the oral subgingival microbiome were assessed by 16S ribosomal DNA (rDNA) sequencing with the Illumina MiSeq platform as previously described^26^. Briefly, bacterial DNA was isolated from subgingival samples (maxillary and mandibular samples pooled) with the DSP Virus/Pathogen Mini Kit in QIAsymphony SP automated system (Qiagen, Valencia, CA). Before DNA extraction, an enzymatic pretreatment was performed for more efficient isolation of Gram-positive bacteria. Metagenomic DNA was subsequently amplified for the 16S rRNA gene hypervariable V3–V4 region with negative (extraction reagents and microbial DNA free water) and positive (subgingival plaque pools and Zymogen mock DNA standard) controls. Three hundred base paired-end sequencing (2 × 300) was performed using the MiSeq Reagent Kit V3 on the Illumina MiSeq. Paired-end sequences were joined using Paired-End read merger (PEAR version 0.9.6). The joined sequences were then filtered for quality with the Fastx-Toolkit (V.0.013) to isolate the Illumina paired-end reads that had 90% of their bases measured up to a score of at least Q30^31^. This score means that only 1 out of every 1000 bases may be incorrect^32^. Only participant samples that had a minimum at least 3,000 reads were included in our analytic sample^31^. Following quality filtering, sequences were clustered at 97% identity against the Human Oral Microbiome Database (HOMD) version 14.5^33^ with Basic Logical Alignment Search Tool (BLAST) aiming at the species level^34^. Finally, in the raw OTU tables, any OTU that had a frequency count of < 0.02% of the total reads was removed from the sample^31^.

### Assessment of additional covariates

Participant characteristics including height, weight, and blood pressure were measured in the OsteoPerio clinic by trained examiners. Either as part of the broader WHI OS^25^ or the OsteoPerio Study^26^, data were collected on women’s age, race/ethnicity, education, medical and oral history, lifestyle and health behaviors, dietary supplement intake, and use of medications, including antibiotics, in the last 30 days.

### Statistical analysis

The subgingival microbiome was analyzed using Compositional Data Analysis techniques^35,36^ to avoid spurious correlations arising from compositional structure in the data. We used the centered log^2^-ratio (CLR) transformation, which represents the abundance of taxa relative to the geometric mean of the sample, and is defined by the formula *CLR*(***x***) = log_2_(***x***/*g*(***x***)), where *g*(***x***) is the geometric mean of the vector ***x***^37^. We added 1 to all counts because of the existence of some zero values. This removes the zeros and keeps proportions of non-zero counts close to their natural values. Since we are using a logarithm base 2, a CLR transformed abundance of 3 represents a species with 2^3^ times greater abundance than the average within the sample. Hereinafter, the CLR transformed relative abundance of each OTU is referred to as “relative abundance”. Measures of relative abundance are considered primary endpoints for this analysis, along with measures of alpha- and beta-diversity.

A correlation matrix across all carbohydrate variables was computed. Mean carbohydrate intake was described by the level of participant characteristics. T-tests and ANOVAs were used to test for significant mean differences across characteristics. We examined the association between carbohydrate intake and three indices of alpha-diversity: observed OTU count, the Chao-1 Index^38,39^ (both representing species richness), and the Shannon Index (representing species evenness)^40,41^. We regressed each alpha-diversity measure on each carbohydrate variable and GL to examine the intra-individual microbial diversity in relation to carbohydrate intake. We also tested differences in the beta-diversity of the microbiome by carbohydrate intake by examining measures of Euclidean distance within and between quartile groups of each carbohydrate intake and GL variable using a PERMANOVA test. We visualized the associations by graphing the samples according to the top two principal components explaining variance in our 245 OTUs, color-coding the points by quartile, and drawing 95% content ellipses.

We also regressed each OTU’s relative abundance measure on continuous measure of total carbohydrate intake, GL, and subtype of carbohydrate. We present crude models and models adjusted for age, race and ethnicity, frequency of flossing, frequency of brushing, frequency of dental visits, smoking status, pack-years of smoking, and antibiotic use. We also considered models further adjusted for body mass index (BMI) and diabetes status, which may be in the causal pathway between carbohydrate intake and the composition of the subgingival microbiome. Data was missing for smoking status (n = 1), frequency of flossing (n = 5), and pack-years of smoking (n = 27) therefore adjusted models have 1,172 rather than 1,204 participants. Crude and adjusted beta-coefficients, associated standard errors, and p-values for each carbohydrate variable and OTU association are presented. The beta-coefficients represent the difference in the relative abundance of a specific OTU for each one-unit increase in carbohydrate/GL intake. A Bonferroni correction for the p-values was used to account for multiple comparisons (0.05 divided by 245). In exploratory analyses, we also repeated our analyses for total carbohydrate intake further adjusted for sucrose. In this way, we explored to what extent the associations with total carbohydrate intake were explained by simple rather than complex carbohydrate intake.

In exploratory analyses, we examined which food groups explained the greatest between person variation in carbohydrate or GL intake. Only carbohydrate variables found to be significantly associated with microbiome relative abundance were examined. We used forward stepwise regression with an inclusion criteria p-value of 0.10 and an exclusion p-value of 0.05 to identify significant contributing foods groups.

## Results

We examined a correlation matrix of all carbohydrate variables and GL. The strongest correlations (≥ 0.70) were seen between total carbohydrates and GL, total fiber, and soluble fiber; between all fiber types (total, soluble, and insoluble); and between fructose and glucose (Supplemental Table 1).

With the exception of antibiotic use, all participant characteristics were associated with at least some of the carbohydrate components (Tables 1 and 2). There was greater mean soluble fiber, fructose, and glucose intake in older compared to younger women. Sucrose intake was highest in Non-Hispanic Black/African Americans and lowest in Hispanic/Latinas. Fructose and glucose intake was highest in Non-Hispanic Black/African Americans and lowest in Non-Hispanic Whites. The mean intake of total carbohydrate, total fiber, soluble fiber, insoluble fiber, and galactose was greater in women with a post-college education compared to those with less education. Intake of total carbohydrates, GL, total fiber, soluble fiber, insoluble fiber, fructose, galactose, and glucose intake was higher in those with a low compared to high BMI. Never-smokers had the highest intake of total carbohydrate, GL, total fiber, soluble fiber, insoluble fiber, fructose, and glucose, followed by former smokers, and current smokers. Dietary sucrose and glucose intake were  lower in women reporting diabetes compared to those with no history of diabetes.

**Table 1:** Mean energy-adjusted total carbohydrate intake, glycemic load, fiber intake, and starch intake by category of participant characteristics (n = 1,204).

Participant characteristic	n	Total carbohydrate (%kcals)	Glycemic load (g/1000)	Total fiber (g/1000)	Soluble fiber (g/1000)	Insoluble fiber (g/1000)	Starch (%kcals)
**Age (years)**							
50–60	234	50.9 (10.5)	65.7 (14.7)	10.5 (3.7)	2.8 (0.9)	7.7 (2.9)	18.7 (4.9)
60–70	551	51.8 (9.3)	66.5 (13.0)	10.9 (3.7)	3.0 (1.0)	7.9 (2.8)	18.9 (4.9)
70–80	387	52.0 (9.1)	66.8 (12.4)	11.1 (3.5)	3.0 (0.9)	8.1 (2.7)	18.4 (4.5)
80 +	32	55.4 (9.1)	72.0 (12.6)	11.9 (4.7)	3.3 (1.2)	8.6 (3.5)	18.4 (4.4)
p-value*		0.085	0.088	0.113	**0.009**	0.223	0.438
**Race**							
American Indian/Alaskan Native	6	49.8 (9.8)	62.9 (14.6)	10.5 (5.1)	2.9 (1.3)	7.6 (3.8)	16.7 (6.8)
Asian/Pacific Islander	3	55.7 (5.0)	69.0 (8.4)	10.2 (1.1)	2.4 (0.3)	7.8 (0.8)	19.1 (1.9)
Non-Hispanic Black/African-American	18	55.8 (10.3)	71.2 (15.1)	12.6 (5.4)	3.4 (1.4)	9.2 (4.2)	17.3 (5.6)
Hispanic/Latina	4	51.6 (8.7)	65.8 (9.3)	10.6 (3.8)	3.3 (0.9)	7.3 (2.9)	20.0 (3.3)
Non-Hispanic White	1173	51.8 (9.5)	66.5 (13.2)	10.9 (3.6)	2.9 (1.0)	7.9 (2.8)	18.7 (4.7)
p-value		0.415	0.581	0.373	0.217	0.431	0.578
**Education**							
High school	251	50.6 (9.3)	66.1 (13.4)	10.4 (3.5)	2.8 (0.9)	7.5 (2.7)	18.9 (4.8)
College	522	51.6 (9.5)	66.4 (13.3)	10.8 (3.6)	2.9 (1.0)	7.8 (2.7)	18.5 (4.6)
Post college	413	52.9 (9.4)	67.3 (12.8)	11.5 (3.8)	3.1 (1.0)	8.4 (2.9)	18.8 (4.8)
p-value		**0.007**	0.411	**0.0003**	**0.008**	**0.0001**	0.406
**Body mass index (kg/m**^**2**^**)**							
< 18.5	16	55.5 (10.0)	72.3 (12.1)	11.8 (4.1)	3.3 (1.1)	8.4 (3.2)	21.1 (6.2)
18.5–25	516	53.1 (9.8)	68.0 (13.5)	11.4 (3.8)	3.1 (1.0)	8.3 (2.9)	18.7 (4.8)
25–30	415	51.2 (8.9)	65.9 (12.5)	10.8 (3.6)	2.9 (0.9)	7.9 (2.8)	18.7 (4.4)
> = 30	257	49.9 (9.3)	64.5 (13.4)	10.0 (3.1)	2.8 (0.9)	7.2 (2.3)	18.3 (5.0)
p-value		** < .0001**	**0.0009**	** < .0001**	**0.0002**	** < .0001**	0.102
**Smoking status**							
Never smoked	635	52.6 (9.3)	67.8 (13.0)	11.1 (3.7)	3.0 (1.0)	8.0 (2.8)	18.8 (4.6)
Former smoker	536	51.2 (9.6)	65.5 (13.3)	10.9 (3.7)	2.9 (1.0)	7.9 (2.8)	18.5 (4.9)
Current smoker	32	46.3 (7.8)	61.8 (12.0)	7.8 (2.3)	2.2 (0.7)	5.6 (1.7)	17.6 (4.5)
p-value		**0.0002**	**0.001**	** < .0001**	** < .0001**	** < .0001**	0.23
**Pack-years of smoking**							
Never smoked	635	52.6 (9.3)	67.8 (13.0)	11.1 (3.7)	3.0 (1.0)	8.0 (2.8)	18.8 (4.6)
Tertile 1	181	52.0 (9.6)	66.1 (13.5)	11.2 (3.8)	3.0 (1.0)	8.2 (2.9)	18.5 (5.3)
Tertile 2	180	50.6 (9.5)	65.0 (13.2)	10.6 (3.5)	2.8 (1.0)	7.7 (2.6)	18.3 (4.3)
Tertile 3	181	50.2 (9.8)	64.6 (13.5)	10.3 (3.7)	2.8 (1.0)	7.5 (3.0)	18.5 (5.0)
p-value		**0.005**	**0.006**	**0.024**	**0.005**	0.05	0.546
**Diabetes**							
No	1148	51.9 (9.4)	66.7 (13.1)	10.9 (3.7)	2.9 (1.0)	7.9 (1.8)	18.6 (4.7)
Yes	56	50.3 (10.6)	64.0 (14.7)	11.5 (3.5)	3.1 (1.0)	8.4 (2.6)	19.3 (6.3)
p-value		0.227	0.129	0.237	0.308	0.233	0.398
**Teeth brushing**							
Not everyday	8	47.8 (5.2)	64.9 (7.9)	9.2 (1.9)	2.5 (0.6)	6.6 (1.4)	19.5 (4.2)
1 × a day	268	50.4 (10.4)	65.1 (15.0)	10.3 (3.7)	2.8 (1.0)	7.5 (2.8)	18.7 (5.3)
2 × a day	661	52.1 (9.2)	67.0 (12.6)	11.0 (3.5)	3.0 (1.0)	8.0 (2.7)	18.6 (4.6)
> 2 × a day	267	52.7 (9.3)	67.1 (12.8)	11.4 (3.9)	3.0 (1.0)	8.3 (3.1)	18.7 (4.7)
p-value		**0.014**	0.221	**0.004**	**0.011**	**0.004**	0.964
**Dental flossing**							
Not every week	217	50.8 (9.5)	66.3 (13.6)	10.3 (3.7)	2.8 (1.0)	7.5 (2.8)	18.7 (4.8)
Once a week	115	50.1 (9.8)	65.2 (13.7)	10.7 (3.6)	2.9 (0.9)	7.8 (2.8)	18.2 (5.3)
> 1 × a week	348	51.0 (9.4)	65.4 (12.9)	10.6 (3.5)	2.9 (1.0)	7.7 (2.7)	18.4 (4.5)
Everyday	519	53.3 (9.4)	67.9 (13.0)	11.4 (3.7)	3.1 (0.9)	8.3 (2.9)	18.9 (4.8)
p-value		** < .0001**	**0.023**	**0.0005**	**0.002**	**0.0006**	0.322
**Dental visits**							
Never	7	48.9 (9.3)	62.1 (13.2)	9.5 (5.4)	2.7 (1.6)	6.8 (3.9)	15.2 (2.3)
Only with a problem	96	49.6 (9.6)	65.1 (13.9)	10.0 (3.6)	2.7 (1.0)	7.2 (2.8)	18.0 (5.2)
Once a year	172	51.9 (9.01)	67.2 (12.6)	10.7 (3.6)	2.9 (1.0)	7.8 (2.7)	19.1 (4.8)
> 1 × a year	929	52.1 (9.6)	66.7 (13.2)	11.1 (3.7)	3.0 (1.0)	8.0 (2.8)	18.7 (4.7)
p-value		0.091	0.491	**0.026**	0.115	**0.021**	0.08
**Antibiotic use**							
No	1081	51.7 (9.5)	66.5 (13.2)	10.9 (3.7)	3.0 (1.0)	7.9 (2.8)	18.6 (4.7)
Yes	123	52.6 (9.8)	67.7 (13.1)	11.0 (3.6)	2.9 (0.9)	8.0 (2.9)	19.0 (5.0)
p-value		0.359	0.332	0.831	0.841	0.727	0.396

*p-value is for the significance of the t-test or ANOVA. Bolded values are statistically significant at a p < 0.05.

**Table 2:** Mean Energy-adjusted Disaccharide and Monosaccharide Intake by Category of Participant Characteristics (n = 1,204).

		Disaccharide Intake			Monosaccharide Intake		
Participant Characteristic	n	Lactose (%kcal)	Maltose (%kcal)	Sucrose (%kcals)	Fructose (%kcals)	Galactose (%kcal)	Glucose (%kcal)
**Age (years)**							
50–60	234	4.8 (3.4)	0.722 (0.3)	8.7 (3.3)	5.5 (2.5)	0.095 (0.1)	5.5 (2.1)
60–70	551	4.9 (3.4)	0.764 (0.3)	8.7 (3.2)	5.7 (2.6)	0.092 (0.1)	5.8 (2.2)
70–80	387	5.0 (3.4)	0.766 (0.3)	8.9 (3.2)	5.9 (2.6)	0.081 (0.1)	6.0 (2.3)
80 +	32	5.1 (2.7)	0.680 (0.3)	10.0 (2.7)	6.8 (2.4)	0.080 (0.1)	6.8 (2.1)
p-value*		0.848	0.15	0.195	**0.025**	0.247	**0.003**
**Race**							
American Indian/Alaskan Native	6	4.7 (3.1)	0.754 (0.3)	7.6 (2.3)	6.2 (3.5)	0.114 (0.1)	6.0 (2.6)
Asian/Pacific Islander	3	7.6 (5.0)	0.475 (0.1)	7.1 (0.9)	7.4 (4.5)	0.118 (0.1)	7.3 (3.1)
Non-Hispanic Black/African-American	18	3.4 (3.1)	0.916 (0.5)	10.8 (5.3)	7.8 (2.9)	0.099 (0.2)	7.4 (1.9)
Hispanic/Latina	4	4.7 (1.7)	0.634 (0.1)	6.2 (2.3)	5.9 (2.5)	0.050 (0.03)	6.0 (1.8)
Non-Hispanic White	1173	4.9 (3.4)	0.753 (0.3)	8.8 (3.2)	5.7 (2.6)	0.088 (0.001)	5.8 (2.2)
p-value		0.224	0.103	**0.022**	**0.0095**	0.851	**0.031**
**Education**							
High school	251	4.7 (3.0)	0.762 (0.3)	8.6 (3.3)	5.4 (2.8)	0.071 (0.1)	5.5 (2.4)
College	522	4.8 (3.4)	0.756 (0.3)	8.9 (3.3)	5.8 (2.7)	0.086 (0.1)	5.9 (2.2)
Post College	413	5.2 (3.6)	0.748 (0.3)	8.9 (3.0)	5.9 (2.4)	0.102 (0.1)	6.0 (2.1)
p-value		0.088	0.853	0.536	0.052	**0.0004**	0.069
**Body mass index (kg/m**^**2**^**)**							
< 18.5	16	4.5 (3.3)	0.798 (0.3)	9.3 (2.9)	6.0 (3.4)	0.137 (0.2)	6.1 (2.9)
18.5–25	516	5.1 (3.7)	0.760 (0.3)	8.9 (3.3)	6.0 (2.5)	0.097 (0.1)	6.1 (2.2)
25–30	415	4.8 (2.8)	0.769 (0.3)	8.8 (3.1)	5.6 (2.5)	0.081 (0.1)	5.7 (2.2)
> = 30	257	4.8 (3.5)	0.717 (0.3)	8.7 (3.3)	5.4 (2.8)	0.081 (0.1)	5.5 (2.3)
p-value		0.491	0.178	0.683	**0.014**	**0.012**	**0.001**
**Smoking status**							
Never smoked	635	4.8 (3.2)	0.760 (0.3)	8.9 (3.0)	6.0 (2.7)	0.090 (0.1)	6.1 (2.3)
Former smoker	536	5.1 (3.6)	0.753 (0.3)	8.7 (3.3)	5.5 (2.4)	0.089 (0.1)	5.6 (2.1)
Current smoker	32	3.8 (2.4)	0.694 (0.2)	9.3 (4.4)	4.6 (2.1)	0.071 (0.1)	4.9 (1.7)
p-value		0.07	0.507	0.341	**< .0001**	0.589	**< .0001**
**Pack-years of smoking**							
Never smoked	635	4.8 (3.2)	0.760 (0.3)	8.9 (3.0)	6.0 (2.7)	0.090 (0.1)	6.1 (2.3)
Tertile 1	181	5.5 (3.9)	0.749 (0.3)	8.5 (2.9)	5.7 (2.5)	0.094 (0.1)	5.8 (2.1)
Tertile 2	180	4.8 (3.5)	0.785 (0.3)	8.8 (3.2)	5.4 (2.4)	0.091 (0.1)	5.6 (2.1)
Tertile 3	181	4.8 (3.3)	0.705 (0.3)	8.9 (4.1)	5.1 (2.3)	0.078 (0.1)	5.3 (2.0)
p-value		0.099	0.095	0.497	** < 0.0001**	0.42	**< 0.0001**
**Diabetes**							
No	1148	4.9 (3.4)	0.756 (0.3)	8.9 (3.2)	5.7 (2.6)	0.089 (0.1)	5.9 (2.2)
Yes	56	5.5 (3.6)	0.728 (0.3)	7.5 (3.0)	5.2 (2.8)	0.081 (0.1)	5.0 (2.0)
p-value		0.162	0.516	**0.001**	0.109	0.573	**0.003**
**Teeth brushing**							
Not everyday	8	4.0 (1.7)	0.684 (0.3)	8.5 (2.9)	4.5 (1.9)	0.051 (0.02)	4.9 (1.8)
1 × a day	268	4.5 (3.3)	0.741 (0.3)	8.5 (3.3)	5.5 (2.9)	0.081 (0.1)	5.6 (2.3)
2 × a day	661	5.0 (3.3)	0.753 (0.3)	8.9 (3.2)	5.7 (2.5)	0.091 (0.1)	5.9 (2.2)
> 2 × a day	267	5.1 (3.6)	0.774 (0.3)	8.9 (3.1)	5.9 (2.5)	0.091 (0.1)	6.0 (2.1)
p-value		0.12	0.587	0.342	0.126	0.389	0.111
**Dental flossing**							
Not every week	217	4.5 (2.9)	0.754 (0.3)	8.9 (3.4)	5.5 (2.7)	0.077 (0.1)	5.6 (2.3)
Once a week	115	4.3 (2.7)	0.747 (0.4)	8.9 (3.4)	5.4(2.5)	0.072 (0.1)	5.6 (2.2)
> 1 × a week	348	4.7 (3.3)	0.750 (0.3)	8.7 (3.0)	5.7 (2.7)	0.084 (0.1)	5.7 (2.3)
Everyday	519	5.3 (3.7)	0.759 (0.3)	8.9 (3.2)	5.9 (2.5)	0.100 (0.1)	6.0 (2.2)
p-value		**0.002**	0.97	0.846	0.107	**0.005**	0.093
**Dental visits**							
Never	7	6.5 (6.5)	0.623 (0.2)	10.5 (4.6)	5.0 (2.9)	0.084 (0.02)	5.1 (2.4)
Only with a problem	96	4.5 (3.2)	0.723 (0.3)	8.7 (3.7)	5.6 (3.1)	0.079 (0.1)	5.5 (2.4)
Once a year	172	4.8 (3.6)	0.760 (0.3)	9.0 (3.5)	5.6 (2.4)	0.092 (0.1)	5.7 (2.2)
> 1 × a year	929	5.0 (3.3)	0.758 (0.3)	8.8 (3.1)	5.8 (2.6)	0.089 (0.1)	5.8 (2.2)
p-value		0.244	0.503	0.462	0.629	0.784	0.335
**Antibiotic use**							
No	1081	4.9 (3.3)	0.751 (0.3)	8.8 (3.2)	5.7 (2.6)	0.088 (0.1)	5.8 (2.2)
Yes	123	5.2 (3.8)	0.788 (0.3)	9.0 (3.3)	5.8 (2.8)	0.091 (0.1)	5.9 (2.4)
p-value		0.421	0.222	0.608	0.804	0.788	0.822

*p-value is for the significance of the t-test or ANOVA. Bolded values are statistically significant at a p < 0.05.

Mean total carbohydrate intake, total fiber, soluble fiber, and insoluble fiber intake were higher in those who brushed more compared to less frequently. Mean total carbohydrate intake, GL, total fiber, insoluble fiber, lactose, and galactose intake were higher in participants who flossed more compared to less frequently. Dental visits were associated with total and insoluble fiber intake, with higher fiber intake in those who had visited the dentist more as compared to less frequently.

There were 122,631 read pairs generated per sample, 120,032 per sample after merging pair-end sequences, 91,165 reads per sample used for OTU-calling, and 86,972 reads per sample that remained in the OTU table. We identified 245 OTUs in the subgingival plaque samples. Firmicutes was the most abundant phylum identified, accounting for more than 45% of reads within the dataset, followed by Bacteroidetes (17.2%) and Fusobacterium (13.5%). The most abundant species identified were *Veillonella dispar* and *Veillonella parvula*, two species from the phylum Firmicutes neither of which ferment carbohydrates.

As intake of total carbohydrate, GL, lactose, and sucrose increased, all three alpha-diversity measures decreased (Table 3), and as starch intake increased, OTU count decreased. Adjustment of the total carbohydrate model for sucrose intake attenuated the associations with alpha-diversity measures (data not shown); however associations were still statistically significant. Microbial beta-diversity was found to be statistically significantly different by quartile of total carbohydrates, fiber (total, soluble, and insoluble), GL, sucrose, and galactose intake (PERMANOVA p < 0.05). Supplemental Fig. 2 plots the associations between the top two OTU principal components among study participants for GL that had the smallest p-value for PERMANOVA.

**Table 3:** Beta-coefficients and standard errors (SE) associated p-values for regression of alpha-diversity measures on carbohydrate intake (n = 1204).

Alpha-diversity	Beta-coefficient (SE) for carbohydrate	Pearson r*	P-value^†^
**Total carbohydrate intake (%kcal)**			
Chao1 Index	− 0.480 (0.105)	− 0.131	** < .0001**
Shannon Index	− 0.008 (0.002)	− 0.098	**0.0007**
Observed OTU	− 0.432 (0.095)	− 0.131	** < .0001**
**Dietary glycemic load (g/1000)**			
Chao1 Index	− 0.340 (0.076)	− 0.128	** < .0001**
Shannon Index	− 0.005 (0.002)	− 0.089	**0.002**
Observed OUT	− 0.308 (0.068)	− 0.129	** < .0001**
**Dietary fiber (g/1000)**			
Chao1 Index	− 0.452 (0.275)	− 0.047	0.101
Shannon Index	− 0.006 (0.006)	− 0.029	0.313
Observed OTU	− 0.443 (0.247)	− 0.052	0.073
**Dietary soluble fiber (g/1000)**			
Chao1 Index	− 1.700 (1.025)	− 0.048	0.098
Shannon Index	− 0.021 (0.022)	− 0.029	0.322
Observed OTU	− 1.59 (0.921)	− 0.050	0.084
**Dietary insoluble fiber (g/1000)**			
Chao1 Index	0.567 (0.359)	− 0.045	0.115
Shannon Index	− 0.007 (0.008)	− 0.028	0.325
Observed OTU	− 0.564 (0.323)	− 0.050	0.081
**Dietary starch (%kcal)**			
Chao1 Index	− 0.407 (0.212)	− 0.055	0.056
Shannon Index	− 0.003 (0.004)	− 0.019	0.513
Observed OTU	− 0.380 (0.191)	− 0.057	**0.046**
**Dietary lactose (%kcal)**			
Chao1 Index	− 0.898 (0.297)	− 0.087	**0.003**
Shannon Index	− 0.019 (0.006)	− 0.086	**0.003**
Observed OTU	− 0.782 (0.267)	− 0.084	**0.004**
**Dietary maltose (%kcal)**			
Chao1 Index	− 3.09 (3.200)	− 0.028	0.335
Shannon Index	− 0.051 (0.067)	− 0.022	0.453
Observed OTU	− 2.77 (2.874)	− 0.028	0.336
**Dietary sucrose (%kcal)**			
Chao1 Index	− 1.47 (0.311)	− 0.135^‡^	** < .0001**
Shannon Index	− 0.022 (0.007)	− 0.097^‡^	**0.0007**
Observed OTU	− 1.22 (0.280)	− 0.124^‡^	** < .0001**
**Dietary fructose (%kcal)**			
Chao1 Index	− 0.398 (0.388)	− 0.030	0.305
Shannon Index	− 0.008 (0.008)	− 0.030	0.299
Observed OTU	− 0.416 (0.349)	− 0.034	0.234
**Dietary galactose (%kcal)**			
Chao1 Index	− 3.74 (9.913)	− 0.011	0.706
Shannon Index	− 0.276 (0.209)	− .0.038	0.186
Observed OTU	− 0.861 (8.904)	− 0.003	0.923
**Dietary glucose (%kcal)**			
Chao1 Index	− 0.697 (0.452)	− 0.044	0.123
Shannon Index	− 0.014 (0.010)	− 0.042	0.142
Observed OTU	− 0.697 (0.406)	− 0.049	0.087

*Correlation coefficients (r) for linear relationship between the carbohydrate variable and alpha-diversity measure.

^†^P-value for ANOVA. Bolded values are statistically significant at a p < 0.05.

We examined continuous intake of total carbohydrates, GL, and carbohydrate subtypes in relation to the relative abundance of all 245 OTUs (Table 4). The beta-coefficients, standard errors, and p-values for each association examined are shown with no adjustment (crude), adjustment for age, race and ethnicity, frequency of flossing, frequency of brushing, frequency of dental visits, smoking status, pack-years of smoking, and antibiotic use (Model 1); and with further adjustment for BMI, and diabetes status (Model 2). In adjusted models, after correction for multiple comparisons, there were significant associations between intake of total carbohydrates, GL, lactose, and sucrose and the relative abundance of at least one OTU. The relative abundance of *Streptococcus mutans* was positively associated with total carbohydrates, GL and sucrose intake in all models. The association between *Streptococcus mutans* and total carbohydrate intake in Model 2 was not statistically significant after further adjustment for sucrose intake (data not shown). We also observed a positive association between GL and *Sphingomonas HOT 006* in Model 1*,* and in Model 2 we observed a positive association between GL and both *Sphingomonas HOT 006* and *Scardovia wiggsiae.* In Models 1 and 2, we observed a negative association between lactose and *Aggregatibacter segnis,* and a negative association between sucrose and both *TM7_[G-1] HOT 3436* and *Leptotrichia HOT 223.* A positive association between sucrose and *Streptococcus lactarius* was observed only in Model 2*.* Results for all dietary carbohydrate variables and OTUs associated at a p-value of < 0.05 are presented in Supplemental Table 2.

**Table 4:** Linear regression of the relative abundance of oral OTUs on carbohydrate intake and glycemic load with beta-coefficients (ß), standard errors (SE), and associated p-value for each dietary variable. (n = 1,204).

Species/OTU	Crude		Model 1*		Model 2^†^		Partial r ^§^
	ß (SE)	p-Value^‡^	ß (SE)	p-Value^‡^	ß (SE)	p-Value^‡^	
**Total carbohydrates (% kcals)**							
*Sphingomonas HOT 006*	0.016 (0.004)	** < .0001**	0.015 (0.004)	0.0003	0.015 (0.004)	0.0004	0.106
*Rothia dentocariosa*	0.037 (0.009)	** < .0001**	0.030 (0.009)	0.001	0.031 (0.009)	0.001	0.096
*Rothia mucilaginosa*	0.033 (0.008)	** < .0001**	0.026 (0.008)	0.002	0.027 (0.008)	0.002	0.093
***Streptococcus mutans***	0.046 (0.012)	**0.0001**	0.051 (0.012)	** < .0001**	0.056 (0.012)	** < .0001**	0.123
*Brevundimonas diminuta*	0.016 (0.004)	0.0003	0.016 (0.005)	0.0006	0.016 (0.005)	0.0005	0.101
**Glycemic load (g/1,000 Kcals)**							
***Streptococcus mutans***	0.039 (0.009)	** < .0001**	0.041 (0.009)	** < .0001**	0.044 (0.009)	** < .0001**	0.138
***Sphingomonas HOT 006***	0.012 (0.003)	** < .0001**	0.012 (0.003)	** < .0001**	0.012 (0.003)	** < .0001**	0.117
*Streptococcus salivarius*	0.024 (0.006)	0.0002	0.022 (0.007)	0.0009	0.024 (0.007)	0.0002	0.098
*Brevundimonas diminuta*	0.012 (0.003)	0.0002	0.012 (0.003)	0.0003	0.012 (0.003)	0.0002	0.107
***Scardovia wiggsiae***	0.024 (0.007)	0.0003	0.026 (0.007)	0.0002	0.028 (0.007)	** < .0001**	0.111
**Total fiber (g/1,000 Kcals)**							
*Actinomyces HOT 171*	0.092 (0.021)	** < .0001**	0.071 (0.022)	0.001	0.065 (0.022)	0.003	0.095
*Ottowia HOT 894*	0.090 (0.023)	** < .0001**	0.080 (0.023)	0.0007	0.084 (0.024)	0.0004	0.100
*Rothia aeria*	0.090 (0.025)	0.0003	0.053 (0.025)	0.036	0.050 (0.025)	0.048	0.062
*Lautropia mirabilis*	0.086 (0.024)	0.0004	0.052 (0.024)	0.034	0.049 (0.025)	0.046	0.063
**Insoluble fiber (g/1,000 kcals)**							
*Actinomyces HOT 171*	0.123 (0.028)	** < .0001**	0.096 (0.029)	0.0009	0.088 (0.029)	0.002	0.098
*Ottowia HOT 894*	0.118 (0.030)	** < .0001**	0.104 (0.030)	0.0007	0.110 (0.031)	0.0004	0.100
*Neisseria elongata*	0.132 (0.036)	0.0003	0.110 (0.037)	0.003	0.111 (0.037)	0.003	0.087
**Lactose (% Kcals)**							
***Aggregatibacter segnis***	− 0.117 (0.028)	** < .0001**	− 0.125 (0.028)	** < .0001**	− 0.124 (0.028)	** < .0001**	− 0.129
**Sucrose (% Kcals)**							
***Streptococcus mutans***	0.201 (0.035)	** < .0001**	0.198 (0.035)	** < .0001**	0.207 (0.035)	** < .0001**	0.163
***TM7_[G-1] HOT 346***	− 0.104 (0.027)	**0.0001**	− 0.105 (0.027)	**0.0001**	− 0.105 (0.027)	**0.0001**	− 0.113
***Leptotrichia HOT 223***	− 0.094 (0.025)	**0.0001**	− 0.098 (0.025)	**0.0001**	− 0.097 (0.025)	**0.0001**	− 0.113
***Streptococcus lactarius***	0.080 (0.021)	0.0002	0.078 (0.022)	0.0003	0.083 (0.022)	**0.0001**	0.106
*Leptotrichia buccalis*	− 0.111 (0.030)	0.0002	− 0.108 (0.030)	0.0003	− 0.109 (0.030)	0.0003	− 0.105
*Streptococcus intermedius*	0.109 (0.030)	0.0003	0.113 (0.030)	0.0002	0.111 (0.030)	0.0002	0.111
*Streptococcus parasanguinis_II*	0.103 (0.028)	0.0003	0.097 (0.028)	0.0005	0.101 (0.028)	0.0003	0.102
*Leptotrichia HOT 498*	− 0.101 (0.029)	0.0004	− 0.107 (0.029)	0.0002	− 0.109 (0.029)	0.0002	− 0.108
*Brevundimonas diminuta*	0.047 (0.013)	0.0004	0.043 (0.013)	0.001	0.043 (0.013)	0.001	0.095
**Galactose (% Kcals)**							
*Leptotrichia goodfellowii*	2.741 (0.720)	**0.0001**	2.352 (0.725)	0.001	2.323 (0.727)	0.001	0.095

*Model 1 adjusted for age, race and ethnicity frequency of flossing, frequency of brushing, frequency of dental visits, smoking status, pack-years of smoking, and antibiotic use. OTUs are ranked for each carbohydrate or glycemic load by the significance of the p-values for the beta regression coefficients in the crude model. Sample size reduced to n = 1,172 for Model 1.

^†^Adjusted for all covariates in Model 1 plus further adjustment for body mass index (BMI) and diabetes status. Sample size stayed at n = 1,172 for Model 2.

^**‡**^P-values are bolded when a p-value is significant after a Bonferroni correction is applied (0.05/245 = 0.0002). In other words, a p-value < 0.0002 will remain statistically significant after an adjustment for multiple comparisons. Only OTUs with p-values for crude models < 0.0005 are shown (no crude models 0.0005 or higher became statistically significant with adjustment).

^§^Partial r for each carbohydrate variable and glycemic load in Model 1.

Exploratory analyses (Supplemental Table 3) identified which food groups, from a list of 122 food groups on the FFQ, explained at least 80% of the variation in total carbohydrate, GL, lactose, and sucrose intake. Twenty-four of 122 food groups were identified. We summarized these foods into the following descriptive groups: (1) grains and baked-goods; (2) starchy vegetables and fruit, and cooked tomatoes; (3) sugary drinks; (4) added sugar, candy, frozen desserts, and pudding-type desserts; (5) and dairy products.

## Discussion

In this analysis of postmenopausal women, we observed that intake of total carbohydrates, GL, starch, lactose and sucrose were negatively associated with the alpha-diversity of our microbiome measures; increased intake was associated with lower intra-individual diversity. We also observed differences in the diversity of the oral microbiome across level of intake of total carbohydrates, fiber, GL, sucrose, and galactose (beta-diversity). Intake of total carbohydrates, GL, and sucrose were positively associated with the relative abundance of *Streptococcus mutans*, a bacteria with an expansion of carbohydrate metabolizing genes^42,43^. We also observed a positive association between GL and the relative abundance of both *Sphingomonas HOT 006* and *Scardovia wiggsiae,* and between sucrose and *Streptococcus lactarius.* We observed a negative association between lactose and *Aggregatibacter segnis, and* between sucrose and both *TM7_[G-1] HOT 346* and *Leptotrichia HOT 223.* To best of our knowledge, this is one of the first epidemiologic studies to examine associations between habitual carbohydrate intake and subgingival, rather than salivary, microbiome samples; we found that carbohydrate intake is associated with the subgingival microbiome.

Minimal research on habitual carbohydrate intake and the oral microbiome has been conducted in humans. In a study in children, there were significant differences in the relative abundance of 18 species from the biofilm of occlusal surfaces by fermentable carbohydrate consumption assessed using an FFQ^23^. They did not identify *Streptococcus mutans* as one of the18 species. They did identify *Aggregatibacter segnis*, which we observed as associated with lactose intake, and *Rothia mucilaginosa,* which we identified as related to total carbohydrate intake in crude analyses. In a study of Danish adults (aged 20 to 81 years), there were no significant differences in salivary bacterial species by intake of energy-adjusted carbohydrates or the proportion of carbohydrates from sugar^22^. In a three week carbohydrate intervention study of 21 athletes, no significant differences in salivary microbial composition were observed^24^.

Our analysis does not fully capture associations between carbohydrate intake and all medias of the oral microbiome because we exclusively examined the microbiome in subgingival plaque samples unlike the previous studies of habitual carbohydrate intake that examined the microbiome of the saliva or biofilm of occlusal surfaces^22–24^. We found no evidence of *Lactobacillus,* known to be associated with caries risk^44^, in our subgingival plaque samples after we filtered out low abundance OTUs. *Lactobacillus*, highly abundant in saliva, is not found as frequently in the subgingival microbiome^45^. Several studies have identified *Streptococcus mutans* in subgingival microbiome samples, similar to our study^46,47^ Carbohydrates are likely accessible to a different composition of bacteria in subgingival, anaerobic conditions, compared to the salivary environment^48^. Despite likely differences in the microbiome of the saliva and subgingival plaque, studies have detected periodontal pathogens in both mediums, concluding that there is some overlap between these two microbiomes^49,50^. Likely the previous studies’ use of salivary or occlusal surface samples, and differences in participants’ ages may explain differences in our results and previous study findings.

As expected, we found a number of associations between sucrose intake and the subgingival microbiome. Sucrose can be broken down into glucose and fructose and taken up by the bacteria, or it can be cleaved inside the bacterial cell by bacterial enzymes^15^. Starches can be broken down by human salivary amylase or by bacterial amylases. Certain *Streptococci* such as *Streptococcus gordonii* and *Streptococcus mitis* can bind amylase to metabolize starch, while other bacteria, such as *Streptococcus mutans,* have enzymes of their own capable of metabolizing starch^15^. Once broken down into simple sugars, sucrose can be transported into the bacterial cell for energy production^15^. Experimental studies show that increasing sugar and fermentable carbohydrate intake increases prevalence of caries^51^ and that frequent sucrose consumption is associated with decreased species diversity, and increased relative abundance of certain *Streptococcus spp.* in the oral biofilm^52^. Our results support the existing evidence that certain fermentable carbohydrates (e.g., sucrose) promote the growth of cariogenic oral bacteria, such as *Streptococcus mutans*^16,53^ We also observed that increased carbohydrate intake was associated with decreased alpha-diversity similar to other studies^23,52,54^.

The association of carbohydrate intake with periodontal disease, rather than caries, is less well studied^17–21^. There is evidence of associations between increased carbohydrate intake and increased gingival bleeding^17,55^ and positive associations between diets high in percent of calories from carbohydrates and rates of periodontal disease^21^. *Leptotrichia spp.,* which we observed to be positively associate with sucrose intake, has been shown to be associated with gingivitis in some studies^12,56^. The other bacteria we identified as associated with carbohydrate intake or GL have not been previously appreciated as contributing to periodontal disease in the literature^12^ or in this cohort^13^.

There is evidence that fiber intake is associated with decreased risk of periodontal disease progression markers^18,20,57^. In a previous study, the oral microbiome (from extracted mice jaws) of mice fed sugar and fiber pellets compared to mice fed sugar pellets alone was lower in *Streptococcus*, *Staphylococcus*, *Lactobacillus*, and *Enterococcus,* as well as greater in alpha-diversity^58^. This suggests fiber consumption may result from mechanical disruption of oral microbiome by fiber. We did not find any significant differences in alpha-diversity or the relative abundance of any of the measured bacterial species by differing fiber intake. It may be that any effect of fiber on the oral microbiome is less important in a cohort of women who frequently brush their teeth.

The relationship between carbohydrate intake and the relative abundance of bacteria is not just defined by whether a certain bacterium has the metabolic capability to utilize a carbohydrate. If two types of sugar are available, some bacteria may preferentially utilize one sugar over the other, as they possess regulatory mechanisms for carbohydrate metabolism^59^. Therefore, we may not see strong relationships with certain types of sugar if both are present and bacteria prefer one over the other. Additionally, bacteria can uptake sugars that have been cleaved by other bacteria or by salivary amylase^60^. Therefore, even if bacteria do not possess the metabolic capability to cleave a certain sugar, they may still be able to utilize its components, which is why we may see a relationship with a certain type of carbohydrate even if the bacteria cannot metabolize it.

We also identified top contributing food sources of total carbohydrate and GL, sucrose, and lactose in a cohort of postmenopausal women. Our findings suggest that attention to dental hygiene should occur after consumption of these foods (e.g., baked goods, added sugar, candy, milk, etc.). This is in alignment with the American Dental Associations’ guidelines on Diet and Nutrition which states “that oral health depends on proper nutrition and healthy eating habits, and necessarily includes avoiding a steady diet of foods containing natural and added sugars, processed starches and low pH-level acids…”^61^. A recent dietary intervention (n = 11 adults, average age 32 years) showed that milk and yogurt consumption, as compared to sucrose intake, resulted in less growth of cariogenic bacteria. Continued research needs to be conducted to better understand the influence of carbohydrate-containing foods, which also contain other nutrients, on the oral microbiome^62^.

Our study has several limitations. Because it was cross-sectional, we cannot make any assumptions about temporality or causality. FFQs, although useful in that they assess habitual dietary intake, are prone to social desirability bias and often underestimate energy intake^63^. We adjusted for energy intake in an attempt to minimize measurement error and underestimation of energy intake^64^. The measure of relative abundance is also not without its shortcomings^65^. Because relative abundance relies on the proportion of the bacteria rather than their absolute number, the measure may induce spurious correlations^65^. However, this limitation is minimized here by adopting Compositional Data Analysis techniques, such as the use of the CLR transformation. Another limitation is that we were unable to examine our oral microbial compositions by anterior versus posterior teeth or by teeth in the upper (maxillary) versus lower (mandibular) jaw arches. This is because we stored plaque samples from all maxillary teeth together and from all mandibular teeth together and then combined these plaque samples prior to sequencing them for bacterial DNA. We did not do an internal assessment of the reliability of our results. We also corrected for multiple testing for 245 OTUs, but did not further correct for multiple testing across our 11 carbohydrate variables and GL.

The age distribution of our participants could be considered a limitation. However, the postmenopausal age range gave us an opportunity to examine these effects in a subpopulation where the association between carbohydrates and the oral microbiome has not been previously studied. Findings may be different in samples with different ages; a broader age group might have allowed for examination of how more varied intake of carbohydrates might affect the oral microbiome over the lifespan.

Despite the limitations of this study, it has important strengths. It is the first study to examine carbohydrate intake and the subgingival microbiome in a sample consisting exclusively of postmenopausal women. We examined many subtypes of carbohydrate, and GL, in order to better understand which carbohydrate components have the strongest associations with the subgingival microbiome. We were able to control our analyses for potential confounding factors including oral hygiene, smoking, and antibiotic use. The selection of our participants into the OsteoPerio study is another strength—they were not selected based on disease status or dietary intake. This would have made our results less generalizable.

In conclusion, our findings suggest that total carbohydrate and GL, as well as intake of the disaccharides sucrose and lactose, are inversely associated with bacteria alpha-diversity in the subgingival microbiome. Furthermore, the beta-diversity of the microbiome varied by total carbohydrates and GL, but also by certain carbohydrate subtypes (sucrose, galactose, and fiber); and we observed that intake of the total carbohydrates, GL, sucrose and lactose to be significantly associated with the relative abundance of specific OTUs estimating bacterial species. Further study of food group intake and dietary patterns will contribute to our understanding of the extent to which the oral microbiome varies in association with carbohydrate consumption and the extent to which these differences are associated with periodontal disease, oral health, and the influence of oral health on systemic health.

## Supplementary Information

Supplementary Information 1.

Supplementary Information 2.

Supplementary Information 3.

Supplementary Information 4.

Supplementary Information 5.

## Data Availability

Data, codebook, and analytic code used in this report may be accessed in a collaborative mode as described on the Women’s Health Initiative website (www.whi.org). Sequence data is also uploaded at the NCBI Sequence Read Archive (SRA) database. The BioProject ID # is PRJNA796273.
