# Supplementary figures and images for: Dietary carbohydrate intake is associated with the subgingival plaque oral microbiome abundance and diversity in a cohort of postmenopausal women
Source: Sci Rep. 2022 Feb 16;12:2643. doi: 10.1038/s41598-022-06421-2 (PMC8850494; doi:10.1038/s41598-022-06421-2)

**Supplemental Figure 1: Flow Diagram for Study Sample**

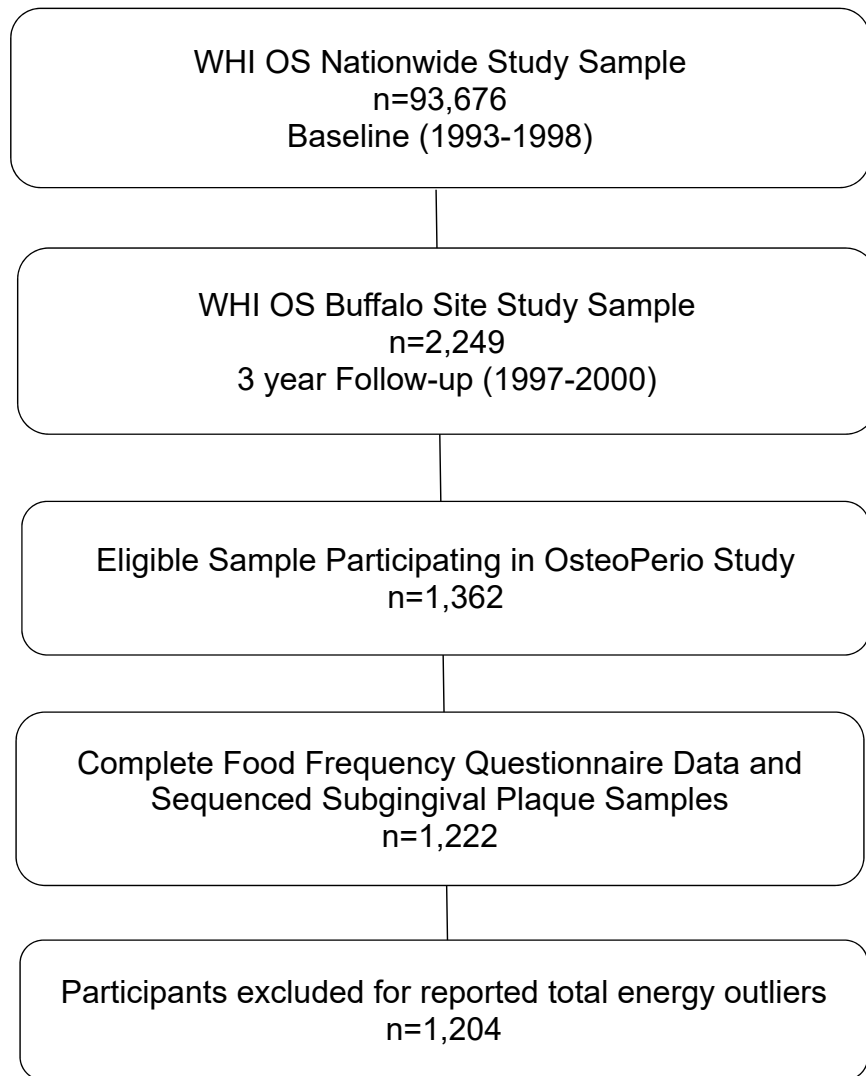

Supplement: Supplementary file 1 — Supplementary Information 1. [file 41598_2022_6421_MOESM1_ESM.pdf]
