# Supplementary material for: Dietary carbohydrate intake is associated with the subgingival plaque oral microbiome abundance and diversity in a cohort of postmenopausal women
Source: Sci Rep. 2022 Feb 16;12:2643. doi: 10.1038/s41598-022-06421-2 (PMC8850494; doi:10.1038/s41598-022-06421-2)

Supplemental Figure 2: Beta-diversity Plot of Subgingival Microbiome Plaque Principal Components 1 and 2 for Glycemic Load (GL).

Legend: This plot shows OTU principal component 1 regressed on principal component 2, and the graphs plot where each participants falls. Woman are color coded for falling into quartile (Q) 1 through 4 for GL. Blue, orange, yellow and pink dots represent women who fall into in Qs 1, 2, 3, and 4, respectively. Circles represent plotted 95% ellipses for each quartile. The p-value from the PERMANOVA test is 0.001 which tests the differences in the beta-diversity of the microbiome by GL by examining measures of Euclidean distance within and between quartile groups of GL.

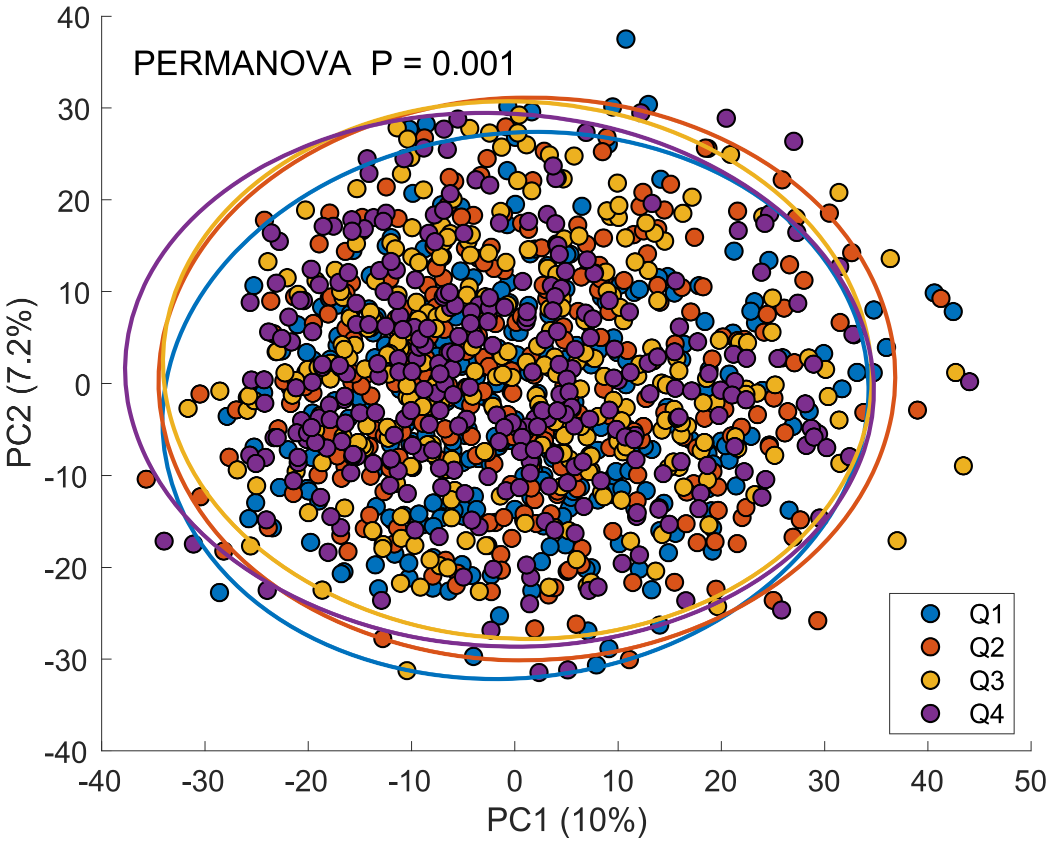

Supplement: Supplementary file 2 — Supplementary Information 2. [file 41598_2022_6421_MOESM2_ESM.pdf]
