# Supplementary material for: Dietary carbohydrate intake is associated with the subgingival plaque oral microbiome abundance and diversity in a cohort of postmenopausal women
Source: Sci Rep. 2022 Feb 16;12:2643. doi: 10.1038/s41598-022-06421-2 (PMC8850494; doi:10.1038/s41598-022-06421-2)
Supplement: Supplementary file 3 — Supplementary Information 3. [file 41598_2022_6421_MOESM3_ESM.docx]

| **Supplemental Table 1:** Pearson Correlation Matrix (r and associated [p-value])* of Dietary Carbohydrates (CHO) and Glycemic Load (n=1,204) | | | | | | | | | | | | |
| --- | --- | --- | --- | --- | --- | --- | --- | --- | --- | --- | --- | --- |
|  | **Total CHO** | **Glycemic Load** | **Total Fiber** | **Soluble Fiber** | **Insoluble Fiber** | **Starch** | **Lactose** | **Maltose** | **Sucrose** | **Fructose** | **Galactose** | **Glucose** |
|  | **% kcals^†^** | **g/1,000 kcals^†^** | | | | **% kcals^†^** | | | | | | |
| Total Carbohydrate | 1 | 0.94 (<.0001) | 0.70 (<.0001) | 0.70 (<.0001) | 0.68 (<.0001) | 0.48 (<.0001) | 0.23 (<.0001) | 0.42 (<.0001) | 0.49 (<.0001) | 0.66 (<.0001) | 0.21 (<.0001) | 0.67 (<.0001) |
| Glycemic Load | 0.94 (<.0001) | 1 | 0.59 (<.0001) | 0.61 (<.0001) | 0.56 (<.0001) | 0.63 (<.0001) | 0.07 (0.0168) | 0.44 (<.0001) | 0.46 (<.0001) | 0.57 (<.0001) | 0.08 (0.009) | 0.59 (<.0001) |
| Total Fiber | 0.70 (<.0001) | 0.59 (<.0001) | 1 | 0.91 (<.0001) | 0.99 (<.0001) | 0.41 (<.0001) | -0.05 (0.0661) | 0.43 (<.0001) | 0.13 (<.0001) | 0.51 (<.0001) | 0.01 (0.7429) | 0.46 (<.0001) |
| Soluble Fiber | 0.70 (<.0001) | 0.61 (<.0001) | 0.91 (<.0001) | 1 | 0.85 (<.0001) | 0.42 (<.0001) | -0.11 (0.0002) | 0.41 (<.0001) | 0.14 (<.0001) | 0.56 (<.0001) | -0.01 (0.6268) | 0.50 (<.0001) |
| Insoluble | 0.68 (<.0001) | 0.56 (<.0001) | 0.99 (<.0001) | 0.85 (<.0001) | 1 | 0.39 (<.0001) | -0.03 (0.3108) | 0.42 (<.0001) | 0.12 (<.0001) | 0.48 (<.0001) | 0.02 (0.5338) | 0.42 (<.0001) |
| Starch | 0.48 (<.0001) | 0.63 (<.0001) | 0.41 (<.0001) | 0.42 (<.0001) | 0.39 (<.0001) | 1 | -0.22 (<.0001) | 0.44 (<.0001) | -0.07 (0.0198) | -0.03 (0.2699) | -0.01 (0.0004) | -0.03 (0.2989) |
| Lactose | 0.23 (<.0001) | 0.07 (0.0168) | -0.05 (0.0661) | -0.11 (0.0002) | -0.03 (0.3108) | -0.22 (<.0001) | 1 | -0.12 (<.0001) | 0.10 (0.0008) | -0.04 (0.1439) | 0.22 (<.0001) | -0.05 (0.1007) |
| Maltose | 0.42 (<.0001) | 0.44 (<.0001) | 0.43 (<.0001) | 0.41 (<.0001) | 0.42 (<.0001) | 0.44 (<.0001) | -0.12 (<.0001) | 1 | 0.14 (<.0001) | 0.14 (<.0001) | 0.05 (0.1167) | 0.18 (<.0001) |
| Sucrose | 0.49 (<.0001) | 0.46 (<.0001) | 0.13 (<.0001) | 0.14 (<.0001) | 0.12 (<.0001) | -0.07 (0.0198) | 0.10 (0.0008) | 0.14 (<.0001) | 1 | 0.19 (<.0001) | 0.34 (<.0001) | 0.23 (<.0001) |
| Fructose | 0.66 (<.0001) | 0.57 (<.0001) | 0.51 (<.0001) | 0.56 (<.0001) | 0.48 (<.0001) | -0.03 (0.2699) | -0.04 (0.1439) | 0.14 (<.0001) | 0.19 (<.0001) | 1 | 0.08 (0.0066) | 0.95 (<.0001) |
| Galactose | 0.21 (<.0001) | 0.08 (0.009) | 0.01 (0.7429) | -0.01 (0.6268) | 0.02 (0.5338) | -0.1 (0.0004) | 0.22 (<.0001) | 0.05 (0.1167) | 0.34 (<.0001) | 0.08 (0.0066) | 1 | 0.09 (0.0013) |
| Glucose | 0.67 (<.0001) | 0.59 (<.0001) | 0.46 (<.0001) | 0.50 (<.0001) | 0.42 (<.0001) | -0.03 (0.2989) | -0.05 (0.1007) | 0.18 (<.0001) | 0.23 (<.0001) | 0.95 (<.0001) | 0.09 (0.0013) | 1 |
| *Pearson correlation coefficients of 0.40 or greater are shaded.  **^†^**Carbohydrate units are the same in the rows and the columns. | | | | | | | | | | | | |
