# Supplementary material for: Dietary carbohydrate intake is associated with the subgingival plaque oral microbiome abundance and diversity in a cohort of postmenopausal women
Source: Sci Rep. 2022 Feb 16;12:2643. doi: 10.1038/s41598-022-06421-2 (PMC8850494; doi:10.1038/s41598-022-06421-2)
Supplement: Supplementary file 4 — Supplementary Information 4. [file 41598_2022_6421_MOESM4_ESM.docx]

| **Supplemental Table 2:** Linear regression of the relative abundance of oral OTUs on total carbohydrate intake with beta-coefficients (ß), standard errors (SE), and associated p-value for each dietary variable (n=1,204).* | | | | | | |
| --- | --- | --- | --- | --- | --- | --- |
| **Species/ OTU** | **Crude** | | **Model 1^†^** | | **Model 2**^‡^ | |
|  | **ß (SE)** | **P-value** | **ß (SE)** | **P-value** | **ß (SE)** | **P-value** |
| ***Total Carbohydrate Intake (%kcal)*** | | | | | | |
| *Rothia mucilaginosa* | 0.033 (0.008) | <.0001 | 0.026 (0.008) | 0.002 | 0.027 (0.008) | 0.002 |
| *Sphingomonas HOT 006* | 0.016 (0.004) | <.0001 | 0.015 (0.004) | 0.0003 | 0.015 (0.004) | 0.0004 |
| *Rothia dentocariosa* | 0.037 (0.009) | <.0001 | 0.030 (0.009) | 0.001 | 0.031 (0.009) | 0.0010 |
| *Streptococcus mutans* | 0.046 (0.012) | 0.0001 | 0.051 (0.012) | <.0001 | 0.056 (0.012) | <.0001 |
| *Brevundimonas diminuta* | 0.016 (0.004) | 0.0003 | 0.016 (0.005) | 0.0006 | 0.016 (0.005) | 0.0005 |
| *Porphyrobacter tepidarius* | 0.013 (0.004) | 0.0006 | 0.012 (0.004) | 0.002 | 0.013 (0.004) | 0.001 |
| *Actinomyces gerencseriae* | 0.027 (0.008) | 0.0007 | 0.024 (0.008) | 0.004 | 0.024 (0.008) | 0.004 |
| *Streptococcus intermedius* | 0.033 (0.010) | 0.0009 | 0.028 (0.010) | 0.007 | 0.026 (0.010) | 0.011 |
| *Microbacterium flavescens* | 0.016 (0.005) | 0.0010 | 0.014 (0.005) | 0.004 | 0.014 (0.005) | 0.004 |
| *Selenomonas HOT 892* | -0.028 (0.009) | 0.001 | -0.029 (0.009) | 0.001 | -0.030 (0.009) | 0.001 |
| *Streptococcus salivarius* | 0.029 (0.009) | 0.001 | 0.026 (0.009) | 0.004 | 0.029 (0.009) | 0.001 |
| *Alloprevotella tannerae* | -0.038 (0.012) | 0.002 | -0.032 (0.013) | 0.010 | -0.034 (0.013) | 0.007 |
| *Actinomyces HOT 171* | 0.025 (0.008) | 0.003 | 0.017 (0.009) | 0.049 | 0.015 (0.009) | 0.077 |
| *Granulicatella adiacens* | 0.018 (0.006) | 0.004 | 0.014 (0.006) | 0.030 | 0.015 (0.006) | 0.024 |
| *Streptococcus parasanguinis_II* | 0.027 (0.010) | 0.005 | 0.027 (0.010) | 0.004 | 0.030 (0.010) | 0.002 |
| *TM7_[G-1] HOT 346* | -0.026 (0.009) | 0.005 | -0.024 (0.009) | 0.011 | -0.024 (0.009) | 0.012 |
| *Veillonella dispar* | 0.018 (0.006) | 0.005 | 0.018 (0.007) | 0.008 | 0.019 (0.007) | 0.004 |
| *Sphingomonas echinoides* | 0.020 (0.007) | 0.006 | 0.019 (0.007) | 0.009 | 0.019 (0.007) | 0.010 |
| *Lachnospiraceae_[G-3] HOT 100* | -0.023 (0.008) | 0.007 | -0.027 (0.009) | 0.002 | -0.029 (0.009) | 0.0009 |
| *Centipeda periodontii* | -0.021 (0.008) | 0.007 | -0.020 (0.008) | 0.011 | -0.020 (0.008) | 0.012 |
| *Scardovia wiggsiae* | 0.025 (0.009) | 0.008 | 0.030 (0.010) | 0.002 | 0.034 (0.010) | 0.0005 |
| *Veillonella parvula* | 0.018 (0.007) | 0.009 | 0.017 (0.007) | 0.021 | 0.018 (0.007) | 0.011 |
| *Peptostreptococcus stomatis* | -0.023 (0.009) | 0.009 | -0.016 (0.009) | 0.075 | -0.016 (0.009) | 0.083 |
| *Haemophilus parainfluenzae* | 0.024 (0.009) | 0.010 | 0.013 (0.009) | 0.173 | 0.011 (0.009) | 0.226 |
| *Selenomonas HOT 146* | -0.022 (0.009) | 0.010 | -0.020 (0.009) | 0.025 | -0.021 (0.009) | 0.019 |
| *Streptococcus parasanguinis_I* | 0.021 (0.008) | 0.010 | 0.015 (0.008) | 0.064 | 0.017 (0.008) | 0.042 |
| *Actinomyces HOT 169* | 0.024 (0.009) | 0.010 | 0.021 (0.010) | 0.027 | 0.023 (0.010) | 0.017 |
| *Streptococcus lactarius* | 0.019 (0.007) | 0.010 | 0.017 (0.007) | 0.023 | 0.019 (0.007) | 0.012 |
| *Aggregatibacter paraphrophilus* | 0.024 (0.009) | 0.011 | 0.019 (0.010) | 0.051 | 0.016 (0.010) | 0.092 |
| *Capnocytophaga HOT 412* | 0.021 (0.009) | 0.012 | 0.021 (0.009) | 0.017 | 0.021 (0.009) | 0.019 |
| *Alloprevotella rava* | -0.021 (0.008) | 0.014 | -0.015 (0.009) | 0.078 | -0.016 (0.009) | 0.071 |
| *Leptotrichia hongkongensis* | 0.024 (0.010) | 0.014 | 0.018 (0.010) | 0.066 | 0.019 (0.010) | 0.055 |
| *Veillonella atypica* | 0.023 (0.009) | 0.015 | 0.022 (0.009) | 0.019 | 0.025 (0.009) | 0.008 |
| *Selenomonas HOT 919* | -0.021 (0.009) | 0.016 | -0.019 (0.009) | 0.036 | -0.019 (0.009) | 0.041 |
| *Leptotrichia HOT 417* | -0.023 (0.010) | 0.016 | -0.025 (0.010) | 0.012 | -0.025 (0.010) | 0.014 |
| *Leptotrichia goodfellowii* | 0.018 (0.008) | 0.019 | 0.011 (0.008) | 0.149 | 0.011 (0.008) | 0.170 |
| *Actinomyces HOT 170* | 0.021 (0.009) | 0.021 | 0.015 (0.009) | 0.107 | 0.013 (0.009) | 0.174 |
| *Campylobacter gracilis* | 0.012 (0.005) | 0.023 | 0.010 (0.005) | 0.063 | 0.010 (0.005) | 0.053 |
| *Tannerella HOT 286* | -0.017 (0.008) | 0.024 | -0.020 (0.008) | 0.009 | -0.022 (0.008) | 0.004 |
| *Prevotella histicola* | 0.021 (0.010) | 0.025 | 0.021 (0.010) | 0.036 | 0.023 (0.010) | 0.019 |
| *Prevotella HOT 292* | 0.020 (0.009) | 0.026 | 0.022 (0.009) | 0.018 | 0.022 (0.009) | 0.017 |
| *Actinomyces massiliensis* | 0.016 (0.007) | 0.028 | 0.007 (0.007) | 0.347 | 0.005 (0.007) | 0.485 |
| *TM7_[G-1] HOT 869* | 0.021 (0.010) | 0.029 | 0.018 (0.010) | 0.075 | 0.015 (0.010) | 0.147 |
| *Fretibacterium HOT 360* | -0.024 (0.011) | 0.032 | -0.013 (0.011) | 0.253 | -0.014 (0.011) | 0.228 |
| *Streptococcus oralis* | 0.012 (0.006) | 0.034 | 0.008 (0.006) | 0.159 | 0.008 (0.006) | 0.175 |
| *Corynebacterium durum* | 0.019 (0.009) | 0.034 | 0.011 (0.009) | 0.192 | 0.008 (0.009) | 0.341 |
| *Prevotella pallens* | -0.017 (0.008) | 0.035 | -0.017 (0.008) | 0.041 | -0.017 (0.008) | 0.041 |
| *Megasphaera micronuciformis* | -0.019 (0.009) | 0.036 | -0.020 (0.009) | 0.027 | -0.020 (0.009) | 0.028 |
| *Selenomonas dianae* | -0.014 (0.007) | 0.037 | -0.010 (0.007) | 0.154 | -0.010 (0.007) | 0.156 |
| *Streptococcus sobrinus* | 0.015 (0.007) | 0.038 | 0.016 (0.007) | 0.034 | 0.017 (0.007) | 0.019 |
| *Leptotrichia HOT 219* | 0.016 (0.008) | 0.043 | 0.008 (0.008) | 0.338 | 0.006 (0.008) | 0.473 |
| *Gemella sanguinis* | 0.014 (0.007) | 0.048 | 0.007 (0.007) | 0.341 | 0.007 (0.007) | 0.328 |
| ***Dietary Glycemic Load (g/1000)*** | | | | | | |
| *Streptococcus mutans* | 0.039 (0.009) | <.0001 | 0.041 (0.009) | <.0001 | 0.044 (0.009) | <.0001 |
| *Sphingomonas HOT 006* | 0.012 (0.003) | <.0001 | 0.012 (0.003) | <.0001 | 0.012 (0.003) | <.0001 |
| *Brevundimonas diminuta* | 0.012 (0.003) | 0.0002 | 0.012 (0.003) | 0.0003 | 0.012 (0.003) | 0.0002 |
| *Streptococcus salivarius* | 0.024 (0.006) | 0.0002 | 0.022 (0.007) | 0.0009 | 0.024 (0.007) | 0.0002 |
| *Scardovia wiggsiae* | 0.024 (0.007) | 0.0003 | 0.026 (0.007) | 0.0002 | 0.028 (0.007) | <.0001 |
| *Porphyrobacter tepidarius* | 0.010 (0.003) | 0.0007 | 0.009 (0.003) | 0.001 | 0.010 (0.003) | 0.0008 |
| *Streptococcus parasanguinis_II* | 0.023 (0.007) | 0.0009 | 0.022 (0.007) | 0.001 | 0.023 (0.007) | 0.0007 |
| *Actinomyces gerencseriae* | 0.019 (0.006) | 0.0010 | 0.017 (0.006) | 0.004 | 0.017 (0.006) | 0.004 |
| *Veillonella dispar* | 0.015 (0.005) | 0.001 | 0.014 (0.005) | 0.002 | 0.016 (0.005) | 0.0010 |
| *Selenomonas HOT 892* | -0.020 (0.006) | 0.001 | -0.020 (0.006) | 0.002 | -0.021 (0.006) | 0.001 |
| *Rothia dentocariosa* | 0.021 (0.007) | 0.001 | 0.019 (0.007) | 0.005 | 0.019 (0.007) | 0.004 |
| *Microbacterium flavescens* | 0.011 (0.003) | 0.002 | 0.010 (0.003) | 0.004 | 0.010 (0.003) | 0.004 |
| *Veillonella atypica* | 0.021 (0.007) | 0.002 | 0.020 (0.007) | 0.003 | 0.022 (0.007) | 0.001 |
| *Lachnospiraceae_[G-3] HOT 100* | -0.018 (0.006) | 0.002 | -0.021 (0.006) | 0.0006 | -0.022 (0.006) | 0.0003 |
| *Streptococcus intermedius* | 0.022 (0.007) | 0.003 | 0.020 (0.007) | 0.008 | 0.018 (0.007) | 0.012 |
| *Rothia mucilaginosa* | 0.017 (0.006) | 0.003 | 0.013 (0.006) | 0.027 | 0.013 (0.006) | 0.028 |
| *Prevotella histicola* | 0.019 (0.007) | 0.007 | 0.017 (0.007) | 0.013 | 0.019 (0.007) | 0.006 |
| *Leptotrichia hongkongensis* | 0.019 (0.007) | 0.008 | 0.016 (0.007) | 0.028 | 0.016 (0.007) | 0.022 |
| *Streptococcus lactarius* | 0.014 (0.005) | 0.010 | 0.012 (0.005) | 0.024 | 0.013 (0.005) | 0.013 |
| *Leptotrichia HOT 417* | -0.018 (0.007) | 0.011 | -0.019 (0.007) | 0.008 | -0.019 (0.007) | 0.009 |
| *Aggregatibacter paraphrophilus* | 0.017 (0.007) | 0.011 | 0.014 (0.007) | 0.039 | 0.013 (0.007) | 0.063 |
| *Alloprevotella tannerae* | -0.022 (0.009) | 0.011 | -0.020 (0.009) | 0.024 | -0.021 (0.009) | 0.017 |
| *Sphingomonas echinoides* | 0.013 (0.005) | 0.011 | 0.013 (0.005) | 0.011 | 0.013 (0.005) | 0.011 |
| *Veillonella parvula* | 0.013 (0.005) | 0.012 | 0.011 (0.005) | 0.030 | 0.012 (0.005) | 0.016 |
| *Selenomonas HOT 146* | -0.015 (0.006) | 0.013 | -0.014 (0.006) | 0.023 | -0.015 (0.006) | 0.019 |
| *Mitsuokella HOT 131* | 0.016 (0.007) | 0.014 | 0.018 (0.007) | 0.006 | 0.020 (0.007) | 0.003 |
| *Selenomonas HOT 919* | -0.015 (0.006) | 0.015 | -0.014 (0.006) | 0.031 | -0.013 (0.006) | 0.039 |
| *Tannerella HOT 286* | -0.013 (0.005) | 0.017 | -0.015 (0.006) | 0.007 | -0.016 (0.006) | 0.004 |
| *Porphyromonas catoniae* | -0.016 (0.007) | 0.017 | -0.018 (0.007) | 0.010 | -0.019 (0.007) | 0.006 |
| *Granulicatella adiacens* | 0.011 (0.005) | 0.019 | 0.009 (0.005) | 0.065 | 0.009 (0.005) | 0.056 |
| *Streptococcus parasanguinis_I* | 0.013 (0.006) | 0.020 | 0.010 (0.006) | 0.099 | 0.011 (0.006) | 0.066 |
| *Leptotrichia HOT 392* | -0.016 (0.007) | 0.021 | -0.016 (0.007) | 0.018 | -0.017 (0.007) | 0.012 |
| *Prevotella HOT 292* | 0.015 (0.006) | 0.022 | 0.015 (0.006) | 0.017 | 0.016 (0.007) | 0.016 |
| *Prevotella pallens* | -0.014 (0.006) | 0.022 | -0.014 (0.006) | 0.023 | -0.013 (0.006) | 0.026 |
| *Capnocytophaga HOT 412* | 0.014 (0.006) | 0.024 | 0.014 (0.006) | 0.029 | 0.014 (0.006) | 0.033 |
| *Peptostreptococcus stomatis* | -0.014 (0.006) | 0.024 | -0.011 (0.007) | 0.088 | -0.011 (0.007) | 0.091 |
| *Actinomyces HOT 169* | 0.015 (0.007) | 0.027 | 0.014 (0.007) | 0.041 | 0.015 (0.007) | 0.027 |
| *Centipeda periodontii* | -0.012 (0.006) | 0.027 | -0.012 (0.006) | 0.042 | -0.012 (0.006) | 0.045 |
| *Porphyromonas HOT 284* | -0.016 (0.007) | 0.028 | -0.016 (0.007) | 0.028 | -0.018 (0.007) | 0.014 |
| *Streptococcus sobrinus* | 0.011 (0.005) | 0.032 | 0.011 (0.005) | 0.041 | 0.012 (0.005) | 0.024 |
| *TM7_[G-1] HOT 346* | -0.014 (0.007) | 0.033 | -0.013 (0.007) | 0.050 | -0.013 (0.007) | 0.054 |
| *Cardiobacterium valvarum* | -0.014 (0.007) | 0.034 | -0.017 (0.007) | 0.009 | -0.020 (0.007) | 0.003 |
| *TM7_[G-1] HOT 488* | -0.016 (0.008) | 0.035 | -0.018 (0.008) | 0.024 | -0.017 (0.008) | 0.034 |
| *TM7_[G-1] HOT 952* | -0.015 (0.007) | 0.043 | -0.015 (0.007) | 0.035 | -0.017 (0.007) | 0.018 |
| *Fretibacterium HOT 360* | -0.016 (0.008) | 0.046 | -0.008 (0.008) | 0.326 | -0.009 (0.008) | 0.290 |
| ***Dietary Fiber (g/1000)*** | | | | | | |
| *Actinomyces HOT 171* | 0.092 (0.021) | <.0001 | 0.071 (0.022) | 0.001 | 0.065 (0.022) | 0.003 |
| *Ottowia HOT 894* | 0.090 (0.023) | <.0001 | 0.080 (0.023) | 0.0007 | 0.084 (0.024) | 0.0004 |
| *Rothia aeria* | 0.090 (0.025) | 0.0003 | 0.053 (0.025) | 0.036 | 0.050 (0.025) | 0.048 |
| *Lautropia mirabilis* | 0.086 (0.024) | 0.0004 | 0.052 (0.024) | 0.034 | 0.049 (0.025) | 0.046 |
| *Neisseria elongata* | 0.098 (0.028) | 0.0005 | 0.080 (0.028) | 0.005 | 0.080 (0.029) | 0.005 |
| *TM7_[G-1] HOT 869* | 0.087 (0.025) | 0.0005 | 0.083 (0.026) | 0.001 | 0.076 (0.026) | 0.003 |
| *Alloprevotella tannerae* | -0.108 (0.031) | 0.0006 | -0.088 (0.032) | 0.007 | -0.089 (0.033) | 0.007 |
| *Corynebacterium durum* | 0.075 (0.023) | 0.0010 | 0.057 (0.023) | 0.012 | 0.051 (0.023) | 0.024 |
| *Veillonella HOT 780* | -0.068 (0.022) | 0.002 | -0.065 (0.022) | 0.004 | -0.061 (0.022) | 0.006 |
| *Rothia mucilaginosa* | 0.064 (0.021) | 0.003 | 0.046 (0.022) | 0.035 | 0.047 (0.022) | 0.032 |
| *Actinomyces massiliensis* | 0.057 (0.019) | 0.003 | 0.029 (0.019) | 0.130 | 0.023 (0.019) | 0.236 |
| *Rothia dentocariosa* | 0.072 (0.024) | 0.003 | 0.051 (0.024) | 0.035 | 0.049 (0.024) | 0.043 |
| *Leptotrichia HOT 219* | 0.059 (0.020) | 0.004 | 0.041 (0.021) | 0.049 | 0.038 (0.021) | 0.073 |
| *Selenomonas dianae* | -0.051 (0.018) | 0.005 | -0.038 (0.018) | 0.036 | -0.037 (0.018) | 0.045 |
| *Streptococcus sanguinis* | 0.056 (0.020) | 0.005 | 0.038 (0.020) | 0.058 | 0.036 (0.020) | 0.075 |
| *Megasphaera micronuciformis* | -0.060 (0.023) | 0.009 | -0.064 (0.024) | 0.007 | -0.068 (0.024) | 0.005 |
| *Selenomonas HOT 146* | -0.057 (0.022) | 0.011 | -0.051 (0.023) | 0.029 | -0.053 (0.023) | 0.023 |
| *Cardiobacterium hominis* | 0.055 (0.022) | 0.012 | 0.034 (0.022) | 0.125 | 0.031 (0.022) | 0.171 |
| *Capnocytophaga gingivalis* | 0.053 (0.021) | 0.013 | 0.023 (0.022) | 0.286 | 0.021 (0.022) | 0.335 |
| *Dialister pneumosintes* | -0.062 (0.025) | 0.014 | -0.026 (0.026) | 0.309 | -0.020 (0.026) | 0.424 |
| *Actinomyces naeslundii* | 0.041 (0.017) | 0.014 | 0.024 (0.017) | 0.161 | 0.021 (0.017) | 0.226 |
| *Actinomyces johnsonii* | 0.048 (0.020) | 0.014 | 0.034 (0.020) | 0.095 | 0.036 (0.020) | 0.077 |
| *Anaeroglobus geminatus* | -0.065 (0.028) | 0.019 | -0.050 (0.028) | 0.072 | -0.048 (0.028) | 0.086 |
| *Selenomonas HOT 936* | -0.051 (0.022) | 0.020 | -0.055 (0.022) | 0.014 | -0.052 (0.023) | 0.021 |
| *Actinomyces gerencseriae* | 0.048 (0.021) | 0.020 | 0.037 (0.021) | 0.084 | 0.033 (0.021) | 0.124 |
| *Selenomonas HOT 136* | -0.054 (0.023) | 0.021 | -0.060 (0.024) | 0.012 | -0.059 (0.024) | 0.015 |
| *TM7_[G-1] HOT 349* | -0.062 (0.027) | 0.022 | -0.046 (0.028) | 0.101 | -0.041 (0.028) | 0.147 |
| *Granulicatella adiacens* | 0.038 (0.017) | 0.024 | 0.027 (0.017) | 0.112 | 0.030 (0.017) | 0.080 |
| *Fretibacterium fastidiosum* | -0.060 (0.027) | 0.025 | -0.019 (0.027) | 0.476 | -0.016 (0.027) | 0.555 |
| *Streptococcus cristatus* | 0.045 (0.020) | 0.025 | 0.030 (0.021) | 0.141 | 0.031 (0.021) | 0.137 |
| *Aggregatibacter aphrophilus* | 0.068 (0.030) | 0.026 | 0.049 (0.031) | 0.118 | 0.047 (0.031) | 0.133 |
| *Prevotella melaninogenica* | -0.050 (0.022) | 0.027 | -0.061 (0.023) | 0.008 | -0.064 (0.023) | 0.006 |
| *Bergeyella HOT 322* | 0.040 (0.018) | 0.029 | 0.023 (0.019) | 0.230 | 0.022 (0.019) | 0.250 |
| *Bacteroidaceae_[G-1] HOT 272* | -0.043 (0.020) | 0.030 | -0.029 (0.020) | 0.144 | -0.023 (0.020) | 0.238 |
| *Tannerella forsythia* | -0.058 (0.027) | 0.031 | -0.039 (0.027) | 0.152 | -0.037 (0.027) | 0.176 |
| *Corynebacterium matruchotii* | 0.042 (0.019) | 0.032 | 0.020 (0.020) | 0.317 | 0.021 (0.020) | 0.306 |
| *Centipeda periodontii* | -0.044 (0.020) | 0.032 | -0.043 (0.021) | 0.041 | -0.042 (0.021) | 0.046 |
| *Actinomyces HOT 170* | 0.050 (0.023) | 0.033 | 0.037 (0.024) | 0.129 | 0.032 (0.024) | 0.188 |
| *Peptostreptococcus stomatis* | -0.049 (0.023) | 0.033 | -0.033 (0.024) | 0.165 | -0.031 (0.024) | 0.193 |
| *Aggregatibacter paraphrophilus* | 0.051 (0.024) | 0.036 | 0.039 (0.025) | 0.114 | 0.032 (0.025) | 0.206 |
| *Microbacterium flavescens* | 0.026 (0.012) | 0.036 | 0.024 (0.013) | 0.058 | 0.025 (0.013) | 0.049 |
| *Streptococcus gordonii* | -0.045 (0.022) | 0.039 | -0.052 (0.022) | 0.019 | -0.047 (0.022) | 0.036 |
| *Selenomonas sputigena* | -0.045 (0.022) | 0.041 | -0.040 (0.022) | 0.076 | -0.037 (0.022) | 0.099 |
| *Capnocytophaga granulosa* | 0.051 (0.025) | 0.041 | 0.029 (0.026) | 0.260 | 0.029 (0.026) | 0.258 |
| *Veillonellaceae_[G-1] HOT 150* | -0.050 (0.025) | 0.042 | -0.030 (0.025) | 0.234 | -0.027 (0.025) | 0.271 |
| *Kingella denitrificans* | 0.049 (0.024) | 0.042 | 0.040 (0.025) | 0.109 | 0.040 (0.025) | 0.110 |
| *Neisseria bacilliformis* | 0.049 (0.025) | 0.046 | 0.055 (0.025) | 0.031 | 0.053 (0.026) | 0.038 |
| *Streptococcus salivarius* | 0.046 (0.023) | 0.047 | 0.035 (0.024) | 0.146 | 0.039 (0.024) | 0.106 |
| *Pseudoramibacter alactolyticus* | -0.044 (0.022) | 0.049 | -0.012 (0.022) | 0.601 | -0.008 (0.022) | 0.724 |
| ***Dietary Soluble Fiber (g/1000)*** | | | | | | |
| *TM7_[G-1] HOT 869* | 0.326 (0.093) | 0.0005 | 0.315 (0.096) | 0.001 | 0.287 (0.096) | 0.003 |
| *Ottowia HOT 894* | 0.297 (0.085) | 0.0005 | 0.270 (0.087) | 0.002 | 0.279 (0.088) | 0.001 |
| *Actinomyces HOT 171* | 0.279 (0.080) | 0.0005 | 0.213 (0.082) | 0.010 | 0.190 (0.083) | 0.022 |
| *Selenomonas dianae* | -0.202 (0.067) | 0.003 | -0.164 (0.068) | 0.016 | -0.164 (0.068) | 0.017 |
| *Lautropia mirabilis* | 0.261 (0.090) | 0.004 | 0.148 (0.091) | 0.105 | 0.137 (0.092) | 0.134 |
| *Corynebacterium durum* | 0.242 (0.084) | 0.004 | 0.198 (0.084) | 0.019 | 0.170 (0.084) | 0.044 |
| *Rothia aeria* | 0.262 (0.094) | 0.005 | 0.154 (0.093) | 0.100 | 0.137 (0.094) | 0.143 |
| *Neisseria elongata* | 0.287 (0.104) | 0.006 | 0.216 (0.106) | 0.043 | 0.211 (0.107) | 0.048 |
| *Rothia dentocariosa* | 0.237 (0.089) | 0.008 | 0.175 (0.090) | 0.052 | 0.165 (0.090) | 0.068 |
| *Alloprevotella tannerae* | -0.310 (0.117) | 0.008 | -0.256 (0.121) | 0.035 | -0.250 (0.122) | 0.040 |
| *Leptotrichia HOT 219* | 0.197 (0.076) | 0.009 | 0.145 (0.078) | 0.063 | 0.132 (0.078) | 0.090 |
| *Capnocytophaga gingivalis* | 0.200 (0.079) | 0.011 | 0.101 (0.080) | 0.208 | 0.087 (0.080) | 0.280 |
| *Actinomyces johnsonii* | 0.184 (0.073) | 0.012 | 0.139 (0.076) | 0.066 | 0.149 (0.076) | 0.050 |
| *Actinomyces gerencseriae* | 0.192 (0.077) | 0.013 | 0.158 (0.079) | 0.047 | 0.141 (0.079) | 0.076 |
| *Megasphaera micronuciformis* | -0.213 (0.085) | 0.013 | -0.228 (0.088) | 0.010 | -0.238 (0.088) | 0.007 |
| *Prevotella melaninogenica* | -0.207 (0.083) | 0.013 | -0.257 (0.085) | 0.003 | -0.259 (0.086) | 0.003 |
| *Rothia mucilaginosa* | 0.193 (0.079) | 0.014 | 0.125 (0.081) | 0.121 | 0.125 (0.081) | 0.123 |
| *Fretibacterium HOT 360* | -0.260 (0.108) | 0.016 | -0.201 (0.110) | 0.068 | -0.196 (0.110) | 0.075 |
| *Anaeroglobus geminatus* | -0.244 (0.103) | 0.017 | -0.212 (0.104) | 0.042 | -0.202 (0.105) | 0.054 |
| *Tannerella forsythia* | -0.236 (0.100) | 0.019 | -0.182 (0.102) | 0.073 | -0.167 (0.102) | 0.103 |
| *Veillonella HOT 780* | -0.190 (0.081) | 0.019 | -0.172 (0.083) | 0.039 | -0.164 (0.084) | 0.050 |
| *Capnocytophaga HOT 412* | 0.193 (0.083) | 0.020 | 0.191 (0.086) | 0.026 | 0.187 (0.086) | 0.030 |
| *Neisseria bacilliformis* | 0.212 (0.091) | 0.020 | 0.258 (0.094) | 0.006 | 0.254 (0.095) | 0.008 |
| *Actinomyces massiliensis* | 0.160 (0.071) | 0.025 | 0.065 (0.071) | 0.366 | 0.045 (0.072) | 0.529 |
| *Granulicatella adiacens* | 0.138 (0.062) | 0.026 | 0.107 (0.063) | 0.087 | 0.110 (0.063) | 0.079 |
| *Streptococcus sanguinis* | 0.166 (0.075) | 0.028 | 0.117 (0.075) | 0.116 | 0.106 (0.075) | 0.160 |
| *Streptococcus cristatus* | 0.163 (0.074) | 0.028 | 0.107 (0.077) | 0.164 | 0.106 (0.077) | 0.169 |
| *Capnocytophaga granulosa* | 0.201 (0.094) | 0.032 | 0.124 (0.095) | 0.195 | 0.121 (0.096) | 0.207 |
| *Fretibacterium fastidiosum* | -0.213 (0.099) | 0.032 | -0.085 (0.100) | 0.399 | -0.062 (0.100) | 0.540 |
| *Cardiobacterium hominis* | 0.172 (0.082) | 0.035 | 0.109 (0.083) | 0.187 | 0.095 (0.083) | 0.253 |
| *Selenomonas HOT 146* | -0.171 (0.083) | 0.039 | -0.150 (0.086) | 0.082 | -0.154 (0.087) | 0.076 |
| *Prevotella HOT 292* | 0.173 (0.085) | 0.042 | 0.183 (0.088) | 0.037 | 0.179 (0.088) | 0.042 |
| *Dialister pneumosintes* | -0.185 (0.093) | 0.047 | -0.064 (0.096) | 0.501 | -0.028 (0.095) | 0.771 |
| *Streptococcus gordonii* | -0.161 (0.081) | 0.048 | -0.182 (0.083) | 0.028 | -0.162 (0.083) | 0.052 |
| *Prevotella pallens* | -0.155 (0.079) | 0.049 | -0.135 (0.082) | 0.098 | -0.152 (0.082) | 0.065 |
| ***Dietary Insoluble Fiber (g/1000)*** | | | | | | |
| *Actinomyces HOT 171* | 0.123 (0.028) | <.0001 | 0.096 (0.029) | 0.0009 | 0.088 (0.029) | 0.002 |
| *Ottowia HOT 894* | 0.118 (0.030) | <.0001 | 0.104 (0.030) | 0.0007 | 0.110 (0.031) | 0.0004 |
| *Rothia aeria* | 0.122 (0.033) | 0.0002 | 0.071 (0.033) | 0.029 | 0.068 (0.033) | 0.037 |
| *Lautropia mirabilis* | 0.116 (0.032) | 0.0003 | 0.070 (0.032) | 0.027 | 0.067 (0.032) | 0.037 |
| *Neisseria elongata* | 0.132 (0.036) | 0.0003 | 0.110 (0.037) | 0.003 | 0.111 (0.037) | 0.003 |
| *Alloprevotella tannerae* | -0.147 (0.041) | 0.0004 | -0.119 (0.042) | 0.005 | -0.121 (0.043) | 0.004 |
| *TM7_[G-1] HOT 869* | 0.109 (0.033) | 0.0008 | 0.104 (0.034) | 0.002 | 0.095 (0.034) | 0.005 |
| *Veillonella HOT 780* | -0.093 (0.028) | 0.0009 | -0.090 (0.029) | 0.002 | -0.085 (0.029) | 0.004 |
| *Corynebacterium durum* | 0.098 (0.030) | 0.0010 | 0.072 (0.030) | 0.015 | 0.066 (0.030) | 0.027 |
| *Actinomyces massiliensis* | 0.078 (0.025) | 0.002 | 0.042 (0.025) | 0.096 | 0.033 (0.025) | 0.185 |
| *Rothia mucilaginosa* | 0.086 (0.028) | 0.002 | 0.063 (0.028) | 0.026 | 0.065 (0.028) | 0.023 |
| *Rothia dentocariosa* | 0.094 (0.031) | 0.003 | 0.066 (0.032) | 0.036 | 0.064 (0.032) | 0.043 |
| *Leptotrichia HOT 219* | 0.077 (0.027) | 0.004 | 0.053 (0.027) | 0.053 | 0.048 (0.027) | 0.078 |
| *Streptococcus sanguinis* | 0.076 (0.026) | 0.004 | 0.051 (0.026) | 0.053 | 0.049 (0.026) | 0.066 |
| *Actinomyces naeslundii* | 0.058 (0.022) | 0.007 | 0.037 (0.022) | 0.098 | 0.033 (0.022) | 0.140 |
| *Selenomonas dianae* | -0.063 (0.023) | 0.008 | -0.046 (0.024) | 0.054 | -0.044 (0.024) | 0.070 |
| *Selenomonas HOT 146* | -0.076 (0.029) | 0.009 | -0.067 (0.030) | 0.026 | -0.071 (0.030) | 0.020 |
| *Cardiobacterium hominis* | 0.074 (0.029) | 0.010 | 0.045 (0.029) | 0.119 | 0.041 (0.029) | 0.161 |
| *Dialister pneumosintes* | -0.083 (0.033) | 0.011 | -0.037 (0.033) | 0.268 | -0.032 (0.033) | 0.339 |
| *Megasphaera micronuciformis* | -0.076 (0.030) | 0.011 | -0.082 (0.031) | 0.008 | -0.087 (0.031) | 0.005 |
| *Selenomonas HOT 936* | -0.072 (0.028) | 0.011 | -0.078 (0.029) | 0.007 | -0.075 (0.029) | 0.011 |
| *TM7_[G-1] HOT 349* | -0.090 (0.035) | 0.012 | -0.068 (0.037) | 0.063 | -0.062 (0.037) | 0.093 |
| *Bergeyella HOT 322* | 0.058 (0.024) | 0.015 | 0.035 (0.025) | 0.154 | 0.035 (0.025) | 0.156 |
| *Capnocytophaga gingivalis* | 0.066 (0.028) | 0.017 | 0.028 (0.028) | 0.322 | 0.026 (0.028) | 0.358 |
| *Aggregatibacter aphrophilus* | 0.094 (0.040) | 0.018 | 0.069 (0.041) | 0.088 | 0.067 (0.041) | 0.102 |
| *Selenomonas HOT 136* | -0.072 (0.030) | 0.018 | -0.079 (0.031) | 0.011 | -0.077 (0.031) | 0.014 |
| *Actinomyces johnsonii* | 0.060 (0.026) | 0.020 | 0.041 (0.027) | 0.126 | 0.043 (0.027) | 0.107 |
| *Actinomyces HOT 170* | 0.070 (0.031) | 0.022 | 0.052 (0.031) | 0.099 | 0.046 (0.032) | 0.142 |
| *Bacteroidaceae_[G-1] HOT 272* | -0.058 (0.026) | 0.023 | -0.040 (0.026) | 0.121 | -0.033 (0.026) | 0.204 |
| *Centipeda periodontii* | -0.060 (0.026) | 0.024 | -0.057 (0.027) | 0.035 | -0.057 (0.027) | 0.038 |
| *Anaeroglobus geminatus* | -0.081 (0.036) | 0.025 | -0.060 (0.036) | 0.098 | -0.058 (0.037) | 0.113 |
| *Aggregatibacter paraphrophilus* | 0.071 (0.032) | 0.026 | 0.055 (0.032) | 0.090 | 0.045 (0.033) | 0.171 |
| *Granulicatella adiacens* | 0.048 (0.022) | 0.028 | 0.033 (0.022) | 0.134 | 0.038 (0.022) | 0.089 |
| *Actinomyces gerencseriae* | 0.059 (0.027) | 0.028 | 0.044 (0.028) | 0.115 | 0.039 (0.028) | 0.160 |
| *Fretibacterium fastidiosum* | -0.076 (0.035) | 0.029 | -0.023 (0.035) | 0.518 | -0.020 (0.035) | 0.572 |
| *Streptococcus cristatus* | 0.057 (0.026) | 0.030 | 0.039 (0.027) | 0.150 | 0.040 (0.027) | 0.141 |
| *Peptostreptococcus stomatis* | -0.065 (0.030) | 0.030 | -0.044 (0.031) | 0.153 | -0.042 (0.031) | 0.177 |
| *Microbacterium flavescens* | 0.035 (0.016) | 0.032 | 0.032 (0.017) | 0.055 | 0.034 (0.017) | 0.042 |
| *Corynebacterium matruchotii* | 0.055 (0.025) | 0.032 | 0.026 (0.026) | 0.324 | 0.027 (0.026) | 0.304 |
| *Streptococcus constellatus* | -0.073 (0.036) | 0.039 | -0.044 (0.036) | 0.226 | -0.038 (0.036) | 0.303 |
| *Kingella denitrificans* | 0.064 (0.031) | 0.041 | 0.053 (0.032) | 0.103 | 0.053 (0.033) | 0.103 |
| *Veillonellaceae_[G-1] HOT 150* | -0.065 (0.032) | 0.041 | -0.038 (0.032) | 0.242 | -0.036 (0.032) | 0.262 |
| *Selenomonas sputigena* | -0.058 (0.029) | 0.045 | -0.050 (0.029) | 0.083 | -0.048 (0.029) | 0.101 |
| *Alloprevotella rava* | -0.058 (0.029) | 0.045 | -0.035 (0.030) | 0.238 | -0.039 (0.030) | 0.192 |
| *Prevotella melaninogenica* | -0.059 (0.029) | 0.045 | -0.072 (0.030) | 0.016 | -0.076 (0.030) | 0.012 |
| *Tannerella forsythia* | -0.071 (0.035) | 0.045 | -0.045 (0.036) | 0.209 | -0.043 (0.036) | 0.227 |
| *Streptococcus salivarius* | 0.061 (0.030) | 0.045 | 0.047 (0.031) | 0.129 | 0.052 (0.031) | 0.094 |
| *Streptococcus gordonii* | -0.057 (0.029) | 0.046 | -0.067 (0.029) | 0.022 | -0.060 (0.029) | 0.040 |
| *Actinomyces HOT 169* | 0.063 (0.032) | 0.047 | 0.049 (0.033) | 0.132 | 0.052 (0.033) | 0.111 |
| ***Dietary Starch (%kcal)*** | | | | | | |
| *Streptococcus mutans* | 0.066 (0.024) | 0.006 | 0.063 (0.024) | 0.010 | 0.066 (0.024) | 0.006 |
| *Corynebacterium matruchotii* | 0.041 (0.015) | 0.006 | 0.033 (0.015) | 0.031 | 0.033 (0.015) | 0.031 |
| *Prevotella intermedia* | -0.063 (0.023) | 0.006 | -0.058 (0.023) | 0.013 | -0.058 (0.023) | 0.014 |
| *Streptococcus salivarius* | 0.049 (0.018) | 0.007 | 0.042 (0.018) | 0.021 | 0.044 (0.018) | 0.014 |
| *Scardovia wiggsiae* | 0.050 (0.019) | 0.008 | 0.053 (0.019) | 0.005 | 0.055 (0.019) | 0.004 |
| *Prevotella HOT 306* | 0.035 (0.014) | 0.014 | 0.034 (0.014) | 0.018 | 0.035 (0.014) | 0.015 |
| *Selenomonas HOT 146* | -0.039 (0.017) | 0.022 | -0.032 (0.018) | 0.071 | -0.031 (0.018) | 0.074 |
| *Atopobium parvulum* | 0.037 (0.016) | 0.025 | 0.034 (0.017) | 0.038 | 0.033 (0.017) | 0.046 |
| *Tannerella forsythia* | -0.046 (0.021) | 0.028 | -0.022 (0.021) | 0.284 | -0.022 (0.021) | 0.296 |
| *Sphingomonas HOT 006* | 0.018 (0.008) | 0.029 | 0.015 (0.008) | 0.064 | 0.015 (0.008) | 0.059 |
| *Capnocytophaga HOT 903* | 0.035 (0.016) | 0.035 | 0.032 (0.017) | 0.060 | 0.032 (0.017) | 0.061 |
| *Tannerella HOT 808* | -0.030 (0.014) | 0.036 | -0.024 (0.015) | 0.102 | -0.024 (0.015) | 0.102 |
| *Leptotrichia HOT 219* | 0.032 (0.016) | 0.040 | 0.024 (0.016) | 0.136 | 0.024 (0.016) | 0.129 |
| *Fretibacterium HOT 360* | -0.045 (0.022) | 0.042 | -0.025 (0.022) | 0.259 | -0.026 (0.022) | 0.252 |
| *Porphyromonas catoniae* | -0.037 (0.018) | 0.047 | -0.044 (0.019) | 0.019 | -0.043 (0.019) | 0.022 |
| *Capnocytophaga HOT 323* | 0.034 (0.017) | 0.047 | 0.035 (0.017) | 0.047 | 0.034 (0.017) | 0.048 |
| *Actinomyces naeslundii* | 0.025 (0.013) | 0.048 | 0.018 (0.013) | 0.172 | 0.016 (0.013) | 0.216 |
| ***Dietary Lactose (%kcal)*** | | | | | | |
| *Aggregatibacter segnis* | -0.117 (0.028) | <.0001 | -0.125 (0.028) | <.0001 | -0.124 (0.028) | <.0001 |
| *Rothia mucilaginosa* | 0.078 (0.023) | 0.0006 | 0.070 (0.023) | 0.002 | 0.070 (0.023) | 0.003 |
| *TM7_[G-1] HOT 346* | -0.082 (0.026) | 0.001 | -0.071 (0.026) | 0.006 | -0.071 (0.026) | 0.006 |
| *Veillonella atypica* | 0.080 (0.026) | 0.002 | 0.074 (0.026) | 0.005 | 0.072 (0.026) | 0.006 |
| *Prevotella histicola* | 0.078 (0.027) | 0.003 | 0.076 (0.027) | 0.005 | 0.075 (0.027) | 0.006 |
| *Streptococcus parasanguinis_II* | 0.077 (0.027) | 0.004 | 0.082 (0.027) | 0.002 | 0.081 (0.027) | 0.002 |
| *Streptococcus parasanguinis_I* | 0.063 (0.022) | 0.005 | 0.056 (0.023) | 0.014 | 0.054 (0.023) | 0.017 |
| *Bergeyella HOT 322* | 0.055 (0.020) | 0.006 | 0.041 (0.020) | 0.043 | 0.042 (0.020) | 0.038 |
| *Veillonella parvula* | 0.053 (0.020) | 0.008 | 0.047 (0.020) | 0.018 | 0.045 (0.020) | 0.025 |
| *Alloprevotella HOT 308* | 0.053 (0.020) | 0.008 | 0.052 (0.020) | 0.010 | 0.051 (0.020) | 0.012 |
| *Prevotella nigrescens* | -0.079 (0.030) | 0.008 | -0.067 (0.031) | 0.028 | -0.068 (0.031) | 0.027 |
| *Prevotella oralis* | 0.075 (0.029) | 0.009 | 0.072 (0.029) | 0.013 | 0.070 (0.029) | 0.016 |
| *Gemella morbillorum* | -0.069 (0.027) | 0.009 | -0.075 (0.027) | 0.005 | -0.076 (0.027) | 0.005 |
| *TM7_[G-6] HOT 870* | -0.060 (0.023) | 0.010 | -0.049 (0.023) | 0.034 | -0.050 (0.023) | 0.030 |
| *Prevotella oris* | -0.068 (0.026) | 0.010 | -0.056 (0.027) | 0.036 | -0.057 (0.027) | 0.032 |
| *Sphingomonas echinoides* | 0.051 (0.020) | 0.011 | 0.046 (0.020) | 0.024 | 0.044 (0.020) | 0.030 |
| *Selenomonas HOT 892* | -0.061 (0.025) | 0.013 | -0.066 (0.025) | 0.009 | -0.065 (0.025) | 0.010 |
| *Actinomyces gerencseriae* | 0.055 (0.022) | 0.014 | 0.046 (0.023) | 0.043 | 0.043 (0.023) | 0.061 |
| *TM7_[G-3] HOT 351* | -0.045 (0.018) | 0.015 | -0.046 (0.019) | 0.014 | -0.047 (0.019) | 0.014 |
| *Rothia dentocariosa* | 0.063 (0.026) | 0.016 | 0.051 (0.026) | 0.047 | 0.049 (0.026) | 0.060 |
| *Desulfobulbus HOT 041* | 0.065 (0.029) | 0.023 | 0.075 (0.029) | 0.009 | 0.078 (0.029) | 0.007 |
| *Pseudoramibacter alactolyticus* | 0.055 (0.024) | 0.024 | 0.068 (0.024) | 0.004 | 0.068 (0.024) | 0.005 |
| *TM7_[G-5] HOT 356* | -0.071 (0.031) | 0.025 | -0.058 (0.032) | 0.069 | -0.056 (0.032) | 0.077 |
| *Porphyrobacter tepidarius* | 0.024 (0.011) | 0.029 | 0.020 (0.011) | 0.070 | 0.020 (0.011) | 0.076 |
| *Sphingomonas HOT 006* | 0.025 (0.011) | 0.030 | 0.018 (0.011) | 0.109 | 0.018 (0.011) | 0.120 |
| *Veillonella dispar* | 0.039 (0.018) | 0.033 | 0.037 (0.019) | 0.049 | 0.035 (0.019) | 0.062 |
| *Leptotrichia HOT 498* | -0.058 (0.027) | 0.034 | -0.055 (0.028) | 0.049 | -0.055 (0.028) | 0.050 |
| *Kingella oralis* | 0.050 (0.024) | 0.037 | 0.029 (0.024) | 0.231 | 0.028 (0.024) | 0.251 |
| *Selenomonas flueggei* | -0.048 (0.024) | 0.042 | -0.058 (0.024) | 0.016 | -0.059 (0.024) | 0.014 |
| *Selenomonas HOT 136* | 0.050 (0.025) | 0.047 | 0.039 (0.026) | 0.129 | 0.037 (0.026) | 0.149 |
| ***Dietary Maltose (%kcal)*** | | | | | | |
| *Alloprevotella tannerae* | -1.231 (0.364) | 0.0008 | -1.251 (0.372) | 0.0008 | -1.295 (0.372) | 0.0005 |
| *Streptococcus salivarius* | 0.785 (0.270) | 0.004 | 0.777 (0.273) | 0.005 | 0.813 (0.272) | 0.003 |
| *Capnocytophaga leadbetteri* | -0.736 (0.281) | 0.009 | -0.830 (0.287) | 0.004 | -0.843 (0.287) | 0.003 |
| *Streptococcus mutans* | 0.926 (0.360) | 0.010 | 1.008 (0.365) | 0.006 | 1.068 (0.363) | 0.003 |
| *Aggregatibacter aphrophilus* | 0.893 (0.352) | 0.011 | 0.797 (0.358) | 0.026 | 0.760 (0.358) | 0.034 |
| *Alloprevotella HOT 308* | -0.446 (0.212) | 0.035 | -0.413 (0.217) | 0.057 | -0.398 (0.217) | 0.067 |
| *Porphyromonas HOT 279* | -0.594 (0.288) | 0.039 | -0.584 (0.293) | 0.046 | -0.587 (0.293) | 0.046 |
| *Haemophilus haemolyticus* | -0.505 (0.254) | 0.047 | -0.455 (0.262) | 0.083 | -0.443 (0.262) | 0.091 |
| *Atopobium HOT 199* | -0.459 (0.232) | 0.048 | -0.422 (0.232) | 0.070 | -0.431 (0.233) | 0.064 |
| ***Dietary Sucrose (%kcal)*** | | | | | | |
| *Streptococcus mutans* | 0.201 (0.035) | <.0001 | 0.198 (0.035) | <.0001 | 0.207 (0.035) | <.0001 |
| *TM7_[G-1] HOT 346* | -0.104 (0.027) | 0.0001 | -0.105 (0.027) | 0.0001 | -0.105 (0.027) | 0.0001 |
| *Leptotrichia HOT 223* | -0.094 (0.025) | 0.0001 | -0.098 (0.025) | 0.0001 | -0.097 (0.025) | 0.0001 |
| *Streptococcus lactarius* | 0.080 (0.021) | 0.0002 | 0.078 (0.022) | 0.0003 | 0.083 (0.022) | 0.0001 |
| *Leptotrichia buccalis* | -0.111 (0.030) | 0.0002 | -0.108 (0.030) | 0.0003 | -0.109 (0.030) | 0.0003 |
| *Streptococcus intermedius* | 0.109 (0.030) | 0.0003 | 0.113 (0.030) | 0.0002 | 0.111 (0.030) | 0.0002 |
| *Streptococcus parasanguinis_II* | 0.103 (0.028) | 0.0003 | 0.097 (0.028) | 0.0005 | 0.101 (0.028) | 0.0003 |
| *Brevundimonas diminuta* | 0.047 (0.013) | 0.0004 | 0.043 (0.013) | 0.001 | 0.043 (0.013) | 0.001 |
| *Leptotrichia HOT 498* | -0.101 (0.029) | 0.0004 | -0.107 (0.029) | 0.0002 | -0.109 (0.029) | 0.0002 |
| *Lachnospiraceae_[G-3] HOT 100* | -0.083 (0.025) | 0.0008 | -0.081 (0.025) | 0.001 | -0.083 (0.025) | 0.0009 |
| *Scardovia wiggsiae* | 0.093 (0.028) | 0.0009 | 0.084 (0.028) | 0.003 | 0.090 (0.028) | 0.001 |
| *Leptotrichia HOT 215* | -0.083 (0.025) | 0.0009 | -0.074 (0.025) | 0.003 | -0.072 (0.026) | 0.005 |
| *TM7_[G-1] HOT 952* | -0.097 (0.029) | 0.001 | -0.092 (0.030) | 0.002 | -0.099 (0.030) | 0.0010 |
| *Peptostreptococcus stomatis* | -0.082 (0.026) | 0.002 | -0.075 (0.027) | 0.005 | -0.078 (0.027) | 0.004 |
| *Veillonella parvula* | 0.064 (0.021) | 0.002 | 0.062 (0.021) | 0.003 | 0.068 (0.021) | 0.001 |
| *Veillonella dispar* | 0.059 (0.019) | 0.002 | 0.058 (0.019) | 0.003 | 0.063 (0.019) | 0.001 |
| *Streptococcus sobrinus* | 0.064 (0.021) | 0.002 | 0.056 (0.021) | 0.009 | 0.062 (0.021) | 0.004 |
| *Prevotella histicola* | 0.084 (0.028) | 0.003 | 0.073 (0.028) | 0.011 | 0.077 (0.029) | 0.007 |
| *Rothia dentocariosa* | 0.080 (0.027) | 0.004 | 0.082 (0.027) | 0.002 | 0.087 (0.027) | 0.001 |
| *Sphingomonas HOT 006* | 0.035 (0.012) | 0.004 | 0.032 (0.012) | 0.007 | 0.032 (0.012) | 0.008 |
| *Veillonella atypica* | 0.078 (0.027) | 0.004 | 0.076 (0.028) | 0.006 | 0.083 (0.028) | 0.003 |
| *Cardiobacterium valvarum* | -0.076 (0.027) | 0.005 | -0.071 (0.027) | 0.009 | -0.075 (0.027) | 0.005 |
| *Veillonella rogosae* | -0.080 (0.029) | 0.005 | -0.077 (0.029) | 0.008 | -0.079 (0.029) | 0.006 |
| *Actinomyces HOT 169* | 0.077 (0.028) | 0.006 | 0.086 (0.028) | 0.002 | 0.091 (0.028) | 0.001 |
| *Porphyrobacter tepidarius* | 0.031 (0.011) | 0.007 | 0.029 (0.012) | 0.013 | 0.029 (0.012) | 0.013 |
| *Porphyromonas HOT 284* | -0.081 (0.030) | 0.007 | -0.082 (0.030) | 0.006 | -0.084 (0.030) | 0.006 |
| *Bacteroidales_[G-2] HOT 274* | -0.079 (0.030) | 0.007 | -0.078 (0.030) | 0.010 | -0.079 (0.030) | 0.009 |
| *Prevotella salivae* | 0.067 (0.026) | 0.010 | 0.060 (0.026) | 0.021 | 0.071 (0.026) | 0.006 |
| *Peptostreptococcaceae_[XI][G-7] [Eubacterium]_yurii_subsps._yur* | -0.076 (0.030) | 0.011 | -0.069 (0.030) | 0.023 | -0.075 (0.030) | 0.014 |
| *Streptococcus salivarius* | 0.067 (0.026) | 0.012 | 0.063 (0.027) | 0.019 | 0.070 (0.027) | 0.009 |
| *Streptococcus anginosus* | 0.084 (0.034) | 0.013 | 0.081 (0.034) | 0.017 | 0.086 (0.034) | 0.012 |
| *Bifidobacterium dentium* | 0.063 (0.026) | 0.016 | 0.064 (0.026) | 0.015 | 0.068 (0.026) | 0.009 |
| *Streptococcus parasanguinis_I* | 0.056 (0.024) | 0.018 | 0.054 (0.024) | 0.024 | 0.057 (0.024) | 0.018 |
| *Neisseria flavescens* | -0.070 (0.030) | 0.018 | -0.062 (0.030) | 0.041 | -0.069 (0.030) | 0.023 |
| *Leptotrichia HOT 392* | -0.065 (0.028) | 0.019 | -0.058 (0.028) | 0.040 | -0.064 (0.028) | 0.023 |
| *TM7_[G-3] HOT 351* | -0.045 (0.019) | 0.021 | -0.045 (0.020) | 0.023 | -0.043 (0.020) | 0.032 |
| *Gemella morbillorum* | -0.065 (0.028) | 0.021 | -0.062 (0.028) | 0.028 | -0.064 (0.028) | 0.023 |
| *Leptotrichia hongkongensis* | 0.067 (0.029) | 0.021 | 0.068 (0.029) | 0.019 | 0.071 (0.029) | 0.015 |
| *Streptococcus gordonii* | 0.057 (0.025) | 0.022 | 0.063 (0.025) | 0.012 | 0.067 (0.025) | 0.007 |
| *Granulicatella adiacens* | 0.043 (0.019) | 0.023 | 0.044 (0.019) | 0.019 | 0.045 (0.019) | 0.018 |
| *Prevotella saccharolytica* | -0.051 (0.023) | 0.028 | -0.044 (0.023) | 0.063 | -0.046 (0.024) | 0.053 |
| *Rothia mucilaginosa* | 0.053 (0.024) | 0.029 | 0.050 (0.024) | 0.041 | 0.049 (0.024) | 0.043 |
| *Campylobacter gracilis* | 0.033 (0.015) | 0.030 | 0.026 (0.016) | 0.091 | 0.030 (0.016) | 0.055 |
| *Mycoplasma salivarium* | 0.046 (0.022) | 0.037 | 0.040 (0.022) | 0.077 | 0.037 (0.023) | 0.101 |
| *Actinomyces gerencseriae* | 0.048 (0.024) | 0.041 | 0.041 (0.024) | 0.089 | 0.047 (0.024) | 0.051 |
| *Selenomonas HOT 892* | -0.051 (0.026) | 0.049 | -0.043 (0.026) | 0.104 | -0.045 (0.026) | 0.089 |
| *Alloprevotella rava* | -0.049 (0.025) | 0.049 | -0.053 (0.026) | 0.038 | -0.056 (0.026) | 0.030 |
| *Microbacterium flavescens* | 0.028 (0.014) | 0.049 | 0.028 (0.014) | 0.053 | 0.028 (0.014) | 0.050 |
| ***Dietary Fructose (%kcal)*** | | | | | | |
| *Actinomyces HOT 180* | -0.081 (0.026) | 0.002 | -0.077 (0.027) | 0.004 | -0.073 (0.027) | 0.007 |
| *Solobacterium moorei* | -0.068 (0.025) | 0.006 | -0.070 (0.026) | 0.007 | -0.066 (0.026) | 0.012 |
| *Megasphaera micronuciformis* | -0.087 (0.032) | 0.007 | -0.105 (0.033) | 0.002 | -0.105 (0.033) | 0.002 |
| *Rothia mucilaginosa* | 0.078 (0.030) | 0.009 | 0.063 (0.031) | 0.038 | 0.062 (0.031) | 0.043 |
| *TM7_[G-1] HOT 869* | 0.092 (0.035) | 0.009 | 0.083 (0.037) | 0.024 | 0.073 (0.037) | 0.045 |
| *Prevotella melaninogenica* | -0.079 (0.032) | 0.013 | -0.089 (0.032) | 0.006 | -0.085 (0.033) | 0.009 |
| *Prevotella HOT 472* | -0.098 (0.039) | 0.013 | -0.082 (0.041) | 0.044 | -0.081 (0.041) | 0.047 |
| *Actinomyces HOT 171* | 0.073 (0.030) | 0.017 | 0.056 (0.031) | 0.074 | 0.053 (0.031) | 0.088 |
| *Haemophilus parainfluenzae* | 0.080 (0.034) | 0.018 | 0.057 (0.034) | 0.096 | 0.053 (0.035) | 0.129 |
| *Veillonella rogosae* | 0.084 (0.035) | 0.018 | 0.060 (0.036) | 0.099 | 0.053 (0.037) | 0.145 |
| *Peptostreptococcaceae_[XI][G-1] [Eubacterium]_infirmum* | -0.056 (0.026) | 0.027 | -0.059 (0.026) | 0.026 | -0.056 (0.026) | 0.033 |
| *Fusobacterium periodonticum* | 0.062 (0.028) | 0.028 | 0.043 (0.029) | 0.137 | 0.040 (0.029) | 0.173 |
| *Fusobacterium HOT 203* | -0.083 (0.039) | 0.034 | -0.088 (0.040) | 0.027 | -0.092 (0.040) | 0.021 |
| *Prevotella pallens* | -0.063 (0.030) | 0.034 | -0.064 (0.031) | 0.040 | -0.063 (0.031) | 0.044 |
| *Leptotrichia goodfellowii* | 0.060 (0.028) | 0.035 | 0.046 (0.029) | 0.113 | 0.044 (0.029) | 0.128 |
| *Prevotella HOT 306* | -0.054 (0.026) | 0.039 | -0.043 (0.027) | 0.107 | -0.041 (0.027) | 0.125 |
| *Prevotella salivae* | -0.064 (0.032) | 0.046 | -0.077 (0.033) | 0.018 | -0.070 (0.033) | 0.032 |
| ***Dietary Galactose (%kcal)*** | | | | | | |
| *Leptotrichia goodfellowii* | 2.741 (0.720) | 0.0001 | 2.352 (0.725) | 0.001 | 2.323 (0.727) | 0.001 |
| *Actinomyces HOT 170* | 2.890 (0.841) | 0.0006 | 2.313 (0.848) | 0.006 | 2.183 (0.849) | 0.010 |
| *Prevotella oulorum* | -2.327 (0.864) | 0.007 | -1.919 (0.872) | 0.028 | -1.871 (0.875) | 0.033 |
| *Capnocytophaga HOT 326* | 2.622 (0.998) | 0.009 | 2.308 (1.011) | 0.023 | 2.110 (1.010) | 0.037 |
| *SR1_[G-1] HOT 874* | 1.732 (0.664) | 0.009 | 1.394 (0.665) | 0.036 | 1.243 (0.665) | 0.062 |
| *Leptotrichia HOT 392* | 2.188 (0.878) | 0.013 | 1.733 (0.879) | 0.049 | 1.645 (0.881) | 0.062 |
| *Streptococcus australis* | 1.749 (0.702) | 0.013 | 1.444 (0.707) | 0.041 | 1.381 (0.710) | 0.052 |
| *Haemophilus haemolyticus* | 1.887 (0.785) | 0.016 | 1.694 (0.803) | 0.035 | 1.636 (0.806) | 0.043 |
| *Actinomyces HOT 180* | -1.600 (0.667) | 0.017 | -1.621 (0.672) | 0.016 | -1.509 (0.673) | 0.025 |
| *Capnocytophaga HOT 332* | 1.671 (0.705) | 0.018 | 1.620 (0.721) | 0.025 | 1.569 (0.724) | 0.030 |
| *Alloprevotella HOT 473* | 1.863 (0.787) | 0.018 | 2.090 (0.800) | 0.009 | 2.026 (0.803) | 0.012 |
| *Microbacterium flavescens* | 1.031 (0.445) | 0.021 | 0.811 (0.446) | 0.069 | 0.764 (0.447) | 0.087 |
| *Gemella haemolysans* | 1.968 (0.855) | 0.022 | 1.905 (0.856) | 0.026 | 1.959 (0.860) | 0.023 |
| *Corynebacterium durum* | 1.869 (0.817) | 0.022 | 1.076 (0.800) | 0.179 | 0.825 (0.798) | 0.302 |
| *Granulicatella elegans* | 1.663 (0.736) | 0.024 | 1.567 (0.737) | 0.034 | 1.550 (0.741) | 0.037 |
| *Streptococcus HOT 074* | 1.553 (0.690) | 0.024 | 1.317 (0.698) | 0.059 | 1.237 (0.701) | 0.078 |
| *TM7_[G-1] HOT 346* | -1.874 (0.849) | 0.027 | -1.545 (0.855) | 0.071 | -1.516 (0.859) | 0.078 |
| *Parvimonas HOT 393* | -2.279 (1.038) | 0.028 | -2.754 (1.040) | 0.008 | -2.782 (1.045) | 0.008 |
| *Selenomonas HOT 936* | -1.661 (0.781) | 0.034 | -1.622 (0.791) | 0.040 | -1.638 (0.794) | 0.039 |
| *Kingella denitrificans* | 1.830 (0.865) | 0.035 | 1.671 (0.871) | 0.055 | 1.622 (0.875) | 0.064 |
| *Selenomonas flueggei* | -1.650 (0.789) | 0.037 | -1.581 (0.793) | 0.047 | -1.471 (0.796) | 0.065 |
| *TM7_[G-5] HOT 356* | -2.174 (1.041) | 0.037 | -1.767 (1.048) | 0.092 | -1.865 (1.049) | 0.076 |
| *Prevotella maculosa* | -1.537 (0.741) | 0.038 | -1.252 (0.746) | 0.094 | -1.284 (0.747) | 0.086 |
| *Aggregatibacter HOT 458* | 1.791 (0.879) | 0.042 | 1.536 (0.884) | 0.083 | 1.527 (0.888) | 0.086 |
| *Leptotrichia HOT 219* | 1.470 (0.733) | 0.045 | 1.038 (0.735) | 0.158 | 0.950 (0.736) | 0.197 |
| *Selenomonas sputigena* | -1.576 (0.790) | 0.046 | -0.899 (0.785) | 0.253 | -0.713 (0.786) | 0.365 |
| *Prevotella melaninogenica* | -1.604 (0.806) | 0.047 | -1.374 (0.811) | 0.091 | -1.237 (0.813) | 0.128 |
| *Lachnoanaerobaculum orale* | -1.283 (0.648) | 0.048 | -1.021 (0.656) | 0.120 | -0.931 (0.656) | 0.156 |
| ***Dietary Glucose (%kcal)*** | | | | | | |
| *Actinomyces HOT 180* | -0.095 (0.030) | 0.002 | -0.092 (0.031) | 0.003 | -0.086 (0.032) | 0.007 |
| *Rothia mucilaginosa* | 0.098 (0.035) | 0.005 | 0.079 (0.036) | 0.027 | 0.078 (0.036) | 0.030 |
| *Haemophilus parainfluenzae* | 0.106 (0.039) | 0.007 | 0.082 (0.040) | 0.040 | 0.076 (0.040) | 0.061 |
| *Prevotella melaninogenica* | -0.098 (0.037) | 0.008 | -0.117 (0.038) | 0.002 | -0.110 (0.038) | 0.004 |
| *Solobacterium moorei* | -0.077 (0.029) | 0.008 | -0.079 (0.030) | 0.009 | -0.070 (0.030) | 0.022 |
| *Prevotella pallens* | -0.087 (0.035) | 0.012 | -0.092 (0.036) | 0.011 | -0.088 (0.036) | 0.015 |
| *Peptostreptococcaceae_[XI][G-1] [Eubacterium]_infirmum* | -0.074 (0.030) | 0.013 | -0.077 (0.031) | 0.013 | -0.075 (0.031) | 0.016 |
| *Prevotella HOT 472* | -0.109 (0.046) | 0.017 | -0.091 (0.047) | 0.055 | -0.089 (0.048) | 0.062 |
| *Veillonella rogosae* | 0.098 (0.041) | 0.017 | 0.073 (0.043) | 0.086 | 0.064 (0.043) | 0.133 |
| *TM7_[G-1] HOT 869* | 0.097 (0.041) | 0.018 | 0.087 (0.043) | 0.041 | 0.075 (0.043) | 0.082 |
| *Fusobacterium HOT 203* | -0.104 (0.045) | 0.022 | -0.112 (0.046) | 0.016 | -0.120 (0.047) | 0.011 |
| *Actinomyces HOT 171* | 0.078 (0.035) | 0.029 | 0.056 (0.036) | 0.125 | 0.054 (0.037) | 0.142 |
| *Alloprevotella rava* | -0.076 (0.036) | 0.036 | -0.066 (0.037) | 0.079 | -0.071 (0.038) | 0.061 |
| *Megasphaera micronuciformis* | -0.079 (0.038) | 0.036 | -0.101 (0.039) | 0.010 | -0.099 (0.039) | 0.012 |
| *Leptotrichia goodfellowii* | 0.069 (0.033) | 0.037 | 0.055 (0.034) | 0.103 | 0.055 (0.034) | 0.106 |
| *Aggregatibacter paraphrophilus* | 0.083 (0.040) | 0.038 | 0.092 (0.041) | 0.025 | 0.081 (0.041) | 0.050 |
| *Streptococcus HOT 074* | 0.064 (0.032) | 0.041 | 0.048 (0.033) | 0.145 | 0.043 (0.033) | 0.196 |
| *Fusobacterium periodonticum* | 0.065 (0.033) | 0.049 | 0.042 (0.034) | 0.217 | 0.037 (0.034) | 0.282 |
| * Only OTUs with p-values for crude models <0.05 are shown.  ^†^Model 1 adjusted for age, race and ethnicity, frequency of flossing, frequency of brushing, frequency of dental visits, smoking status, pack-years of smoking, antibiotic use. Sample size reduced to n=1,172 for Model 1.  ^‡^Adjusted for all covariates in Model 1 plus further adjustment for body mass index (BMI), diabetes status. Sample size stayed at n=1,172 for Model 2. | | | | | | |
