# Supplementary material for: Dietary carbohydrate intake is associated with the subgingival plaque oral microbiome abundance and diversity in a cohort of postmenopausal women
Source: Sci Rep. 2022 Feb 16;12:2643. doi: 10.1038/s41598-022-06421-2 (PMC8850494; doi:10.1038/s41598-022-06421-2)
Supplement: Supplementary file 5 — Supplementary Information 5. [file 41598_2022_6421_MOESM5_ESM.docx]

| **Supplemental Table 3:** Foods explaining at least 80% of the variance in intake of total carbohydrates, glycemic load, lactose and sucrose in this sample of post-menopausal women (n=1,204) | | | | | | | |
| --- | --- | --- | --- | --- | --- | --- | --- |
| **Total carbohydrate** | | **Glycemic load** | | **Lactose** | | **Sucrose** | |
| **Food** | **Model^*^ R^2^** | **Food** | **Model R^2^** | **Food** | **Model R^2^** | **Food** | **Model R^2^** |
| Dark bread | 0.15 | Dark bread | 0.15 | Milk, all types,  not on cereal | 0.78 | Chocolate candy  and candy bars | 0.24 |
| White breads | 0.25 | White breads | 0.30 | Non-fat yogurt  (not frozen) | 0.86 | Non-fat yogurt  (not frozen) | 0.42 |
| Milk on cereal  (cold & cooked) | 0.35 | Cold cereal | 0.41 |  |  | Sugar in coffee/ tea & on cereal | 0.58 |
| Non-fat yogurt  (not frozen) | 0.42 | Regular soft drinks (not diet) | 0.48 |  |  | Doughnuts, cakes, pastries, Pop-Tarts, or pan dulce | 0.68 |
| Milk, all types,  not on cereal | 0.48 | Cooked cereals & grits | 0.54 |  |  | Hard candy, jams, jelly, honey or syrup | 0.75 |
| Regular soft drinks (not diet) | 0.53 | Biscuits, muffins, scones, and croissants | 0.58 |  |  | Low-fat or non-fat frozen desserts | 0.80 |
| Rice, grains & plain noodles | 0.58 | Rice, grains & plain noodles | 0.62 |  |  |  |  |
| Hard candy, jams, jelly, honey or syrup | 0.62 | Hard candy, jams, jelly, honey or syrup | 0.66 |  |  |  |  |
| Doughnuts, cakes, pastries, Pop-Tarts, or  pan dulce | 0.65 | Milk, all types, not on cereal | 0.69 |  |  |  |  |
| Tomatoes cooked, sauce/salsa/ picante | 0.68 | Doughnuts, cakes, pastries, Pop-Tarts, or pan dulce | 0.72 |  |  |  |  |
| Low-fat or non-fat frozen desserts | 0.70 | Bananas | 0.74 |  |  |  |  |
| Other yogurt  (not frozen) | 0.72 | Cookies | 0.76 |  |  |  |  |
| Cookies | 0.74 | Saltines, SnackWell's, or fat-free chips | 0.78 |  |  |  |  |
| Orange or grapefruit juice | 0.75 | Tomatoes cooked, sauce/ salsa/picante | 0.79 |  |  |  |  |
| Sweet potatoes and yams | 0.77 | Sugar in coffee/ tea & on cereal | 0.81 |  |  |  |  |
| Ice cream | 0.78 |  |  |  |  |  |  |
| Other fruit juices | 0.80 |  |  |  |  |  |  |
| **^*^** Model R^2^ is the multiple R^2^ for the model as each variable is sequentially added. | | | | | | | |
